# ACO/ARO/AIO-22 – External beam radiotherapy combined with endorectal high-dose-rate brachytherapy in elderly and frail patients with rectal cancer: A prospective multicentre trial of the German Rectal Cancer Study Group

**DOI:** 10.1016/j.ctro.2025.100958

**Published:** 2025-04-13

**Authors:** Hendrik Dapper, Maximilian Fleischmann, Nikolaos Tselis, Markus Diefenhardt, Ralf-Dieter Hofheinz, Christian Weiß, Gerhard G. Grabenbauer, Ricarda Merten, Anca-Ligia Grosu, Simon Kirste, Stefan Rieken, Johannes Claßen, Christian Langer, Thomas Kuhnt, Heinz Schmidberger, Michael Ghadimi, Frank Giordano, Ursula Nestle, Stefan A. Koerber, Felix Bock, Matthias Geiger, Bülent Polat, Christiane J. Bruns, Georg Dieplinger, Felix Popp, Thomas Zander, Thomas Brunner, Silke Tribius, Dirk Arnold, Georg Wurschi, Pompiliu Piso, Tim Friede, Juliane Hörner-Rieber, Eleni Gkika, Claus Rödel, Emmanouil Fokas, on behalf of the German Rectal Cancer Study Group

**Affiliations:** a Department of Radiation Oncology, Cyberknife and Radiation Therapy, Faculty of Medicine and University Hospital of Cologne, University of Cologne, Cologne, Germany; b Department of Radiation Oncology, University Hospital Johann Wolfgang Goethe University, Frankfurt, Germany; c Frankfurt Cancer Institute, Frankfurt, Germany; d German Cancer Research Center (DKFZ), Heidelberg, Germany; e Mannheim Cancer Center, University Hospital Mannheim, Mannheim, Germany; f Department of Radiotherapy and Oncology, Community Hospital, Darmstadt, Germany; g Department of Radiation Oncology and Radiotherapy, DiaCura & Klinikum Coburg, Coburg, Germany; h Department of Radiation Therapy, University of Erlangen-Nürnberg, Erlangen, Germany; i Department of Radiation Oncology, Medical Center University of Freiburg, Faculty of Medicine, Freiburg, Germany; j German Cancer Research Center, German Cancer Consortium, Freiburg, Germany; k Department of Radiation Oncology, Comprehensive Cancer Center Lower Saxony (CCC-N), University Hospital Göttingen (UMG), Göttingen, Germany; l Department of Radiation Oncology, St. Vincentius-Kliniken gAG, Karlsruhe, Germany; m Kempten Clinic, Kempten, Germany; n Department of Radiation Therapy, University of Leipzig, Leipzig, Germany; o Department of Radiation Oncology and Radiation Therapy, University Medical Centre of the Johannes Gutenberg University Mainz, Mainz, Germany; p Department of General, Visceral and Pediatric Surgery, University Medical Center, Göttingen, Germany; q Department of Radiation Oncology, University Hospital Mannheim, Mannheim, Germany; r Department of Radiation Oncology, Kliniken Maria Hilf, Moenchengladbach, Germany; s Department of Radiation Oncology, Barmherzige Brüder Hospital Regensburg, Regensburg, Germany; t Department of Radiotherapy and Oncology, University of Rostock, Rostock, Germany; u Department of Radiotherapy and Oncology, Ostfildern Medius Kliniken, Ostfildern, Germany; v Department of Radiation Oncology, University of Würzburg, Würzburg, Germany; w Department of Visceral Surgery, University of Cologne, Cologne, Germany; x Department of Internal Medicine, Center for Integrated Oncology Aachen Bonn Cologne Düesseldorf, University Hospital of Cologne, Cologne, Germany; y Department of Radiation Oncology, Medical University of Graz, Graz, Austria; z Asklepios Tumorzentrum Hamburg, AK St. Georg, Hermann-Holthusen Institute for Radiotherapy, Hamburg, Germany; aa Asklepios Tumorzentrum Hamburg, AK Altona, Department of Oncology and Hematology, Hamburg, Germany; ab Department of Radiotherapy and Radiation Oncology, Jena University Hospital, Jena, Germany; ac Department of General Surgery, Krankenhaus Barmherzige Brüder Regensburg, Regensburg, Germany; ad Department of Medical Statistics, University Medical Center Göttingen, Göttingen, Germany; ae Department of Radiation Oncology, University Hospital Düsseldorf, Germany; af Department of Radiation Oncology, University Hospital Bonn, Bonn, Germany

## Abstract

**Purpose:**

Rectal cancer disproportionately affects the elderly population, with more than half of cases diagnosed in individuals aged 70 years or older. Frail patients in this group often face significant challenges tolerating the standard treatment of total mesorectal excision (TME), due to elevated risks of surgical complications and perioperative morbidity. Advances in radiotherapy techniques offer a viable alternative, providing effective tumor control while minimizing treatment-related toxicity. Recent clinical trials, including OPERA and MORPEUS, have demonstrated that dose-escalated radiotherapy, which integrates external beam (chemo)radiotherapy (EBRT) with high-dose-rate endorectal brachytherapy (HDR-BT) or contact X-ray brachytherapy (CXB), can achieve high clinical response rates and facilitate organ preservation in younger, healthier patient cohorts. Building on these findings, the ACO/ARO/AIO-22 study seeks to adapt these innovative approaches to meet the needs of elderly and frail patients with rectal cancer.

**Methods/Design:**

The ACO/ARO/AIO-22 trial is a prospective multicentre controlled trial. Elderly (age ≥70 years) and/or frail patients with non-metastatic rectal adenocarcinoma (cT1-3d N0/+ M0, mrCRM − / +) localized 0–16 cm from the ano-cutaneous line, unable to undergo radical surgery can be included. The initial treatment comprises an external beam radiation (EBRT) regime with 13 × 3 Gy (total: 39 Gy) over a period of two and a half weeks. Following initial restaging 6.5 weeks after completion of EBRT, endorectal HDR-BT will be delivered with 3 weekly fractions of 8 Gy to a total dose of 24 Gy (prescribed at the radial margin of the tumor; with a maximum prescription depth of 10 mm); alternatively, with CXB with 90 Gy in 3 weekly fractions. The primary objective is complete or near complete clinical response (cCR or ncCR) and the second primary endpoint is quality of life (QoL) measured with the EORTC QLQ-ELD14, both at 12 months after treatment start.

**Discussion:**

The ACO/ARO/AIO-22 prospective multicentre trial will evaluate organ preservation rates and QoL after combining EBRT with endorectal HDR-BT in elderly and/or frail patients with rectal cancer.

**Trial Registration:**Clinicialtrials.gov number: NCT06729645.

## Highlights

• A novel trial combining EBRT with HDR-BT for elderly, frail rectal cancer patients.
• Prioritizes organ preservation and quality of life in a vulnerable patient cohort.
• Uses advanced radiotherapy techniques and planning including brachytherapy to ensure high efficacy with minimal toxicity.
• Focuses on a frequently underrepresented patient group in clinical studies.
• Employs a robust multicenter design ensuring high generalizability and validity of results.

## Background

Approximately half of rectal cancer cases occur in individuals aged 70 or older, a demographic expected to expand considerably in the coming years [1]. For elderly and frail individuals, standard treatments like radical surgery pose substantial risks, including higher rates of surgical complications and mortality due to age and comorbidities [[2], [3], [4], [5], [6], [7], [8]]. A study conducted by the Colorectal Cooperative Group UK (1988–2000), which analyzed 34,194 colorectal cancer patients aged 75 years and older, revealed that 21 % of patients aged over 85 did not undergo surgical resection. In comparison, the rate of non-surgical management was 11 % among patients aged 75–84 years [9]. Additionally, a lack of treatment often leads to disease progression, impacting both quality of life (QoL) and overall outcomes [[10], [11], [12], [13], [14], [15]]. It is essential to adopt a more individualized approach for elderly and frail patients, taking into account factors like the degree of frailty, individual preferences, and specific treatment objectives to ensure the most suitable therapeutic strategy for this expanding patient population.

Recent advancements in radiotherapy, such as intensity-modulated radiotherapy (IMRT) and endoluminal brachytherapy, offer non-invasive alternatives for patients unfit for surgery, with or without neoadjuvant chemotherapy and/or chemoradiation (CRT), or even a total neoadjuvant therapy (TNT) regimen [[16], [17], [18], [19]]. Dose-escalated, high-precision radiotherapy constitutes an attractive option for this vulnerable patient cohort as it can provide long-term local control of the primary tumor, prevent disease-related morbidity, prolong survival and improve QoL [20].

Recent findings from randomized studies (OPERA, MORPEUS) involving younger and healthier rectal cancer patients indicate that increased RT doses can be safely delivered with external beam (chemo)radiotherapy (EBRT) followed by endoluminal radiotherapeutic technics, such as high-dose-rate brachytherapy (HDR-BT) or contact X-ray brachytherapy (CXB) and lead to high remission rates [21,22]. The OPERA trial enrolled 141 rectal cancer patients with cT2-T3a/b N0-1 diseases, characterized by a tumor diameter of less than 5 cm, involvement of no more than half of the rectal circumference and a location within 10 cm of the anal verge. The control group received CRT, consisting of external beam radiotherapy (EBRT) at a dose of 45 Gy delivered in 25 fractions over 5 weeks, along with concurrent capecitabine and an additional EBRT boost of 9 Gy in 5 fractions over 1 week. In contrast, the experimental group underwent the same CRT protocol but with a CXB boost (90 Gy in 3 fractions over 4 weeks), administered either prior to CRT for tumors smaller than 3 cm or afterward for larger tumors. Organ preservation after 3 years was 60 % vs 81 % (p = 0.005) in the control and experimental group, respectively, providing phase III evidence for the efficacy of CXB in patients with relatively small tumors [21].

Furthermore, high remission rates contribute to sustained local control and provide opportunities for organ preservation, along with improved anorectal function and QoL. Haak et al. evaluated the efficacy of a watch-and-wait (W&W) strategy in a cohort of 43 elderly patients who achieved a clinical complete response (cCR) or near complete response (ncCR) following neoadjuvant therapy. After a minimum follow-up of 2 years, 88 % of patients sustained a cCR, and the 3-year OS rate reached 97 % [23]. A further multicentre analysis in 258 patients treated with short course RT combined with CXB showed cCR rates of 70 %) organ preservation rates in 81 %, and stoma-free survival in 94 % of cases after median follow-up of 24 months, supporting its effectiveness as a non-surgical treatment in predominantly elderly patients [24].

However, prospective studies specifically examining this therapeutic approach in elderly and frail rectal cancer patients remain scarce. The proposed ACO/ARO/AIO-22 trial seeks to address this gap by assessing the effectiveness of combining external beam radiotherapy (EBRT) with endorectal brachytherapy to promote organ preservation and sustain QoL in this vulnerable patient population.

## Design/Methods

### Setting

The ACO/ARO/AIO-22 trial is a prospective multicentre investigator-initiated trial study of the German Rectal Cancer Study Group (GRCSG) testing efficiency and tolerability combining EBRT with endorectal HDR-BT in elderly and/or frail patients with a primary diagnosis of non-metastatic rectal cancer. Inclusion criteria (Table 1) are as follows: Elderly patients of all sex (age ≥ 70 years) with life expectancy ≥ 6 months and histologically confirmed diagnosis of rectal adenocarcinoma localized 0–16 cm from the ano-cutaneous line as measured by rigid rectoscopy as well as MRI-defined cT1-3d N0/+ M0, mrCRM − / +, involvement of ≤ 2/3 of the rectal circumference can be included if they have a G8-frailty score ≤ 14 based on the G8 geriatric assessment tool and/or a American Society of Anesthesiologists Physical Status (ASA PS) ≥ 3 and/or are unsuitable to radical surgery as judged by the surgeon and/or with a G8-frailty score ≥ 14 based on the G8 geriatric assessment tool of frailty and/or elderly patients that refuse surgery. Staging requires high-resolution magnetic resonance imaging (MRI) of the pelvis with thin slices (approximately 3 mm) for local assessment, along with spiral computed tomography (CT) scans of the abdomen and chest to rule out distant metastases. Table 1 summarizes the inclusion and exclusion criteria. The G8 geriatric assessment tool and the ASA PS are shown in the Appendix Tables 1 and 2, respectively.

**Table 1:** Inclusion & Exclusion Criteria.

Inclusion criteria	• Informed consent of the patient • Elderly patients (age ≥ 70 years) with a G8-frailty score ≤ 14 based on the G8 geriatric assessment tool of frailty *and/or* elderly patients (age ≥ 70 years) with American Society of Anesthesiologists Physical Status (ASA PS) ≥ 3 *and/or* elderly patients (age ≥ 70 years) unsuitable to tolerate radical surgery as judged by the surgeon *and/or* elderly patients (age ≥ 70 years) with a G8-frailty score ≥ 14 based on the G8 geriatric assessment tool of frailty that refuse radical surgery and/or elderly patients that refuse surgery • Life expectancy ≥ 6 months • Male and female patients with histologically confirmed diagnosis of rectal adenocarcinoma localized 0–16 cm from the anocutaneous line as measured by rigid rectoscopy • MRI-defined cT1-3d N0/+ M0, mrCRM − / +, involvement of ≤ 2/3 of the rectal circumference • Staging requirements: High-resolution, thin-sliced (i.e. 3 mm) magnetic resonance imaging (MRI) of the pelvis is the mandatory local staging procedure. • Spiral-CT of the abdomen and chest to exclude distant metastases.
Exclusion criteria	• cT4 (N any), independent of tumor location • >2/3 involvement of the rectal wall circumference • Distant metastases (to be excluded by CT scan of the thorax and abdomen) • Prior antineoplastic therapy for rectal cancer • Prior radiotherapy of the pelvic region • On-treatment participation in a clinical study in the period 30 days prior to inclusion • Previous or current drug abuse • Other concomitant antineoplastic therapy • Prior or concurrent malignancy ≤ 3 years prior to enrolment in study (Exception: non-melanoma skin cancer or cervical carcinoma FIGO stage 0–1), unless the patient is continuously disease-free • Psychological, familial, sociological or geographical condition potentially hampering compliance with the study protocol and follow-up schedule (these conditions should be discussed with the patient before registration in the trial).

### Primary and secondary objectives

The trial has two primary endpoints. The first primary endpoint is the rate of cCR or ncCR at 12 months after treatment start (i.e. 6 months after first restaging post-treatment). The determination of remission rates (cCR, ncCR, poor/non response) is based on the Maastricht/NKI definition [25] and provided in Table 2. The second primary endpoint is QoL at 12 months after treatment start as assessed by the EORTC QLQ-ELD14 questionnaire that was developed for elderly cancer patients [26].

**Table 2:** Definition of cCR, near cCR and poor response.

**Modality**	**cCR**	**Near cCR**	**Poor response**
**DRE**	No palpable tumor	Small and smooth mucosal irregularity	Palpable tumor mass
**Rectoscopy**	Flat, white scar with *or* without telangiectasiaNo ulcerNo nodules (biopsy not mandatory)	Residual ulcer *or* Small mucosal nodules *or* minor mucosal abnormalities.Mild persisting erythema of the scar	Visible macroscopic tumor
**Pelvic MRI**	Substantial downsizing No residual tumor *or* residual fibrosis only (with limited signal on DWI)Sometimes associated with residual wall thickening due to oedemaNo suspicious lymph nodes	Obvious downstaging with residual fibrosis but heterogeneous *and/or* Irregular aspects and signal, or regression of lymph nodes with no malignant enhancement features, but with a size of > 5 mm	No tumor egression *or*No regression of suspicious lymph nodes.

Abbreviations: cCR, clinical complete response; DRE, digital rectal examination; DWI, diffusion-weighted imaging; MRI, magnetic resonance imaging.

*Table 2 adapted from the international consensus on organ preservation by Fokas et al. with permission [25].

Secondary endpoints include general oncological QoL (EORTC-CR30, −CR29) and anorectal functional outcome (Wexner-Score), G8 geriatric assessment tool, sustained cCR at 2 years, disease-free survival (DFS), cumulative incidence of locoregional regrowth after cCR at 2 years, cumulative incidence of distant metastases at 2 years, OS at 2 years, acute and late toxicity assessment according to National Cancer Institute − Common Terminology Criteria for Adverse Events (NCI CTCAE V.5.0). Exploratory endpoints will be considered in translational/biomarker studies.

If elderly/frail patients have poor response (less than ncCR) or even tumor progression, and will be considered operable at restaging 6 months after treatment initiation or in case of tumor regrowth (despite initially found to be unfit for surgery or in case they refused surgery), then the following secondary endpoints will also be evaluated: Rate of surgery (LE/TME with or without APR/stoma) after poor response/tumor progression at 6 months after treatment initiation, rate of surgery (LE/TME with or without APR/stoma) after locoregional regrowth, cumulative incidence of local recurrence after surgery, postoperative complications of surgery, rate of sphincter-sparing surgery, pathological TNM-staging of surgery, R0 resection rate; negative circumferential resection rate after surgery, tumor regression grading according to Dworak after surgery, neoadjuvant rectal score after surgery, quality of TME surgery according to MERCURY and DFS according to the international consensus on appropriate endpoints for multimodal rectal cancer treatment, also incorporating the W&W approach [27].

### Treatment schedule

**Fig. 1:**
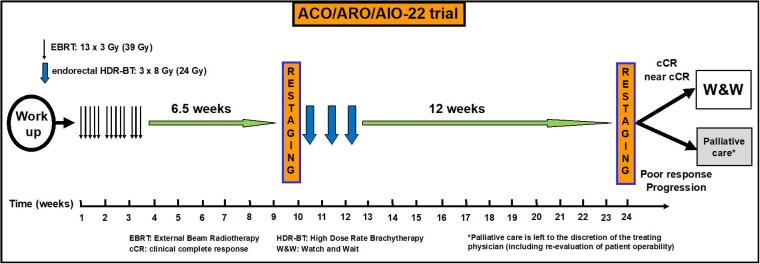
Overview of the treatment schedule of the ACO/ARO/AIO-22 clinical trial.

The treatment schedule is shown in Fig. 1. Treatment recommendation of EBRT is based on the previous studies in elderly and/or frail patients [28,29]. Treatment will be administered according to the following schedule: EBRT with 13 × 3 Gy (total: 39 Gy), 5 fractions per week, over a period of two and a half weeks (time interval: week 1–3). A risk-adapted clinical target volume will be used, as described in detail in the standard operating procedure (SOP) for EBRT in the Appendix. Restaging will be performed with pelvic MRI and endoscopy 6.5 weeks after completion of EBRT and prior to the first endorectal HDR-BT fraction to evaluate therapy response and the residual extent of the disease for target outlining (time point: week 10). After first restaging, endorectal HDR-BT will be delivered with 3 × 8 Gy to a total dose of 24 Gy (prescribed at the radial margin of the tumor; with a maximum prescription depth of 10 mm), with each brachytherapy application performed once weekly over a period of three weeks (time point: week 10–12). A detailed step-by-step and planning SOP for HDR-BT is provided in the Appendix**.**

Upon completion of HDR-BT, restaging with pelvic MRI and endoscopy will be conducted 12 weeks after the last HDR-BT application (timepoint: week 24). Regarding response assessment at restaging, cCR, ncCR, and poor/no response are defined based on clinical examination (digital rectal examination, DRE), pelvic MRI, and endoscopy (Table 2). Depending on tumor response, patients will then be stratified as follows: Patients with cCR or ncCR at 24 weeks (6 months) after start of treatment will be followed up using a standardized W&W program (Table 3). Patients with poor response or, even, tumor progression at 24 weeks (6 months) after start of treatment will be offered palliative care according to the discretion of the treating physician, including re-evaluation of the patient operability in elderly/frail patients. In case re-evaluation indicates that the patient is now operable (even if initially considered as unfit for surgery), then TME or local excision will be performed according to the discretion of the surgeon. In this case, follow-up will be conducted as summarized in the Appendix.

**Table 3:** Follow-up schedule for patients observed within the Watch & Wait program.

**Months after Watch & Wait**	**3**	**6**	**9**	**12**	**15**	**18**	**21**	**24**	**30**	**36**	**48^1^**	**60^1^**
Physical examination, tumor marker CEA	X	X	X	X	X	X	X	X	X	X	X	X
Colonoscopy*		X*										X
Abdomen sonography		X		X		X		X		X	X	X
Rectoscopy	X	X	X	X	X	X	X	X	X	X	X	X
Pelvic MRI	X	X	X	X		X		X		X	X	X
Chest X-ray				X				X		X	X	X

* if a colonoscopy was not performed before surgery (cf. Appendix); next colonoscopy in 5 years in cases of normal findings (lack of adenoma or carcinoma).

### General & response assessment, efficacy evaluation and follow-up procedures

Table 4 provides a comprehensive overview of all study assessment procedures to be conducted and documented. A more intensive local follow-up regimen is recommended for patients to optimize the early detection of local tumor regrowth, as detailed in Table 3. In case full adherence to this intensified follow-up regime is not possible in elderly patients due to frailty, then information on tumor status, patients QoL, frailty and toxicity should be documented at 6, 12 and 24 and 36 months after treatment completion.

**Table 4:** Study assessments.

	**Screening**	**External beam radiotherapy (EBRT)**			**First Restaging:** **week 10** **(tumor assessment 6.5 weeks after EBRT)**	**Endorectal** High-Dose Rate Brachytherapy (HDR-BT)*			**Second Restaging:** **week 24** **(tumor assessment 12 weeks after HDR-BT) ^9^**	**4**–**8 weeks** **after decision for Watch & Wait or palliative treatment** **(or salvage surgery, if performed)**	**Follow-up^10^**
**Assessment**	**1**–**6 weeks before treatment start**	Week 1**	Week 2**	Week 3**		Week 10**	Week 11**	Week 12**			
Informed consent	X										
Medical history	X										
Concomitant diseases/treatment^1^	X	X	X	X		X	X	X			
Physical examination (symptomatic)	X	X	X	X	X	X	X	X	X	X	X
Digital rectal examination	X				X				X		X
ECOG Performance status, weight	X				X				X	X	X
ASA physical status classification	X								X		
Rigid rectosigmoidoscopy	X^7^										X^11^
Endoluminal sonography	X^8^										X^11^
Complete colonoscopic examin.	X^13^										X^11^
Pelvic MRI and flexible rectoscopy^2^	X				X				X		X^11^
CT (thorax, abdomen)^3^	X^13^										X^11^
Hematology (differential)^4^	X								X		
Clinical chemistry^5^	X								X		
Tumor marker CEA	X^13^								X^14^		X
Surgical findings, pathology									X***		
Toxicity/ adv. events		X	X	X	X	X	X	X	X	X	X
Questionnaire life quality, Wexner score ^6^	X								X		X^12^
G8 geriatric assessment tool	X								X		X^12^

1. Specified supportive treatment only.

2. Trans-rectal endoscopic ultrasound (EUS) is additionally used when MRI is not definitive to diagnose early cT1 disease.

3. For exclusion of abdominal or lung metastases.

4. Hematology: Hb, platelets, WBC, neutrophils, lymphocytes.

5. Clinical chemistry: sodium, potassium, calcium, creatinine, urea, bilirubin, GOT, GPT, LDH, alkaline phosphatase, total protein, albumin

6. EORTC QLQ-C30, −CR29, −ELD14, Wexner-Score.

7. Incl. biopsy/histology (biopsy max 60 days old at enrollment into trial).

8. When MRI is not definitive to exclude early cT1 disease in the lower third of the rectum.

9. Within one week for tumor assessment at week 24.

10. For non-operative management (organ preservation), use the Watch & Wait follow-up schedule (Table 3).

11. If clinically indicated according to the follow-up (in case of W&W) and Appendix (in case of salvage surgery).

12. At follow up 6, 12, 24 and 36 months.

13. Baseline assessments of complete colonoscopic examination, CT (thorax, abdomen) and tumor marker CEA may be older than 6 weeks after communication with the Coordinating Investigator.

14. An elevated CEA level, as a solitary finding, will not be considered acceptable evidence of rectal cancer recurrence/progression

* Instead of HDR-BT, CXB with 90 Gy in 3 fractions (30 Gy/fraction) can also be used as performed in the OPERA trial^88^.

** A deviation of treatment days of +/− 4 working days is acceptable.

*** If elderly/frail patients have poor tumor response or progress and will be considered operable at 6 months after treatment initiation or in case of tumor regrowth (despite initially found unfit for surgery), then surgical findings/pathology will be documented (Type of surgery, Clavien-Dindo classification of postoperative morbidity, pathology results (ypTNM staging, tumor regression grading, resection margin [R0/1/2], CRM status, TME quality according to MERCURY criteria.).

### Sample size and justification

The sample size calculation is driven by the primary endpoint alive and cCR or ncCR at 12 months after start of treatment. A sample size of 77 patients yields a power of 80 % at a one-sided significance level α of 5 % to test the hypothesis that the proportion of patients alive with cCR or ncCR at 12 months will be higher than 40 % given that the true proportion is 54 %. Since this is an early clinical trial the one-sided level of 5 % is justified [30]. The estimated 40 % cCR or ncCR rate at 12 months was derived from previous clinical trials. The primary endpoint cCR or ncCR and alive is assessed 1 year after treatment start; it accounts for deaths already and dropout is otherwise unlikely. The sample size is adjusted for about 4 % dropout in this elderly and frail population, which results in a targeted number of in total 80 patients to be enrolled.

The second primary endpoint is QoL at 12 months after treatment start as assessed by the EORTC QLQ-ELD14 questionnaire that was developed for elderly patients with cancer. As long as at least 70 patients are alive and able to complete the questionnaire at 12 months, a one-sample *t*-test comparing the mean changes from baseline to 12 months to 0 has a power of 80 % at a one-sided significance level α of 5 % given a standardized treatment difference of 0.3. Sample size calculations were carried out using nQuery 9 (version 9.2.0.0).

### Statistical aspects

All details of the statistical analysis will be presented in a statistical analysis plan (SAP), which will include definitions of the analysis populations and handling of missing data. Summary statistics such as means, standard deviations and quantiles for continuous data and frequencies (proportions) for categorical data will be provided to describe the patient baseline characteristics such as age, gender, clinical tumor category, clinical nodal category and distance of the tumor from the anal verge.

Local control rate defined as cCR or ncCR at 12 months after treatment start is calculated for the whole patient population, both for the patient group that did not achieve a (n)cCR and underwent palliative treatment (or radical surgery, if considered operable) and patients who achieved a (n)cCR and underwent a W&W approach. Pearson-Clopper confidence intervals (CI) will be calculated for the proportion of patients alive and achieving (n)cCR at 12 months. If the two-sided 90 % CI does not include 40 %, the primary hypothesis can be rejected. For the second primary endpoint, change in QLQ-ELD14 from baseline to 12 months, the mean difference with CI will be reported. The second primary endpoint will only be tested if the first primary endpoint was statistically significant (hierarchical order of hypotheses).

## Discussion

The hereby proposed ACO/ARO/AIO-22 prospective trial will aim to evaluate the efficacy of EBRT in combination with endorectal HDR-BT to achieve organ preservation and maintain QoL in elderly and frail patients with rectal cancer The ACO/ARO/AIO-22 trial incorporates several aspects that make this prospective study important: (1) Treatment decision-making in elderly and frail patients with rectal cancer is particularly challenging due to the lack of standardized guidelines for this population, highlighting the need for clinical trials to establish optimal treatment approaches. Importantly, in this vulnerable group, QoL considerations are essential, as comorbidities and reduced functional reserves often limit tolerance to intensive treatments [31].; (2) the combination of EBRT and HDR-BT has demonstrated high rates of tumor response with almost 90 % overall response and cCR rates of approximately 50–80 %, especially in young and fit patients with early stage tumors, whereas prospective trials in elderly and frail patients remain limited; (3) due to its very steep dose gradient, endorectal HDR-BT enables the delivery of very high radiation doses to the tumor with a very steep fall-off in adjacent normal tissue, thus increasing treatment efficacy while keeping toxicity low. Neoadjuvant external beam radiotherapy in elderly rectal cancer patients has been shown to be feasible and safe [32]. Moreover, short-course radiotherapy is associated with less acute toxicity compared to CRT [33,34]. The efficacy of alternative EBRT regimens, such as 13 × 3 Gy, in achieving local tumor control is currently being explored. The Lion trial investigated whether escalating preoperative radiotherapy improves tumor response and sphincter preservation in low rectal cancer (T2/T3). In 88 patients, adding a CXB boost to standard EBRT significantly increased complete response rates (24 % vs. 2 %), near-complete sterilization (57 % v 34 %) and sphincter preservation (76 % vs. 44 %, P = 0.004) without affecting morbidity or survival at 35 months [28]. These alternative regimens are presently being studied, primarily in conjunction with dose-escalating endoluminal brachytherapy boosts [28,35].

Dose-response investigations have shown that higher radiotherapy doses are associated with improved response rates [36]. Specifically, analyses suggest that doses ≥ 92 Gy (equivalent dose in 2 Gy per fraction [EQD2]) are necessary to achieve a pathological complete response (pCR) in 50 % of patients. Higher radiotherapy doses can be effectively administered using endoluminal brachytherapy techniques, such as CXB or HDR-BT [20,[37], [38], [39], [40], [41], [42], [43]]. Even with CXB alone, patients with stage I rectal cancer can achieve favourable outcomes, including a cCR rate of 82 % and 3-year local control of 84 %, with low toxicity, as shown in a retrospective multicentre study of 76 patients [44]. Various studies have already shown that combined EBRT followed by HDR-BT is very effective and usually well tolerated. Vuong et al. demonstrated a pCR in 32 out of 47 rectal cancer patients (68 %) with cT2-T4 disease who received an HDR-BT boost delivering a total dose of 26 Gy (administered in four fractions over four consecutive days) prior to surgery [42]. In the HERBERT-I trial, 38 elderly and frail patients, with a median age of 83 years, underwent treatment consisting of EBRT (39 Gy in 3 Gy single doses) followed by HDR-BT (3 fractions of 5–8 G). For brachytherapy, the 100 %-isodose was prescribed to a depth of up to 20 mm from the surface of the applicator [35,45]. Patients diagnosed with rectal cancer at stage cT2-4 N0-1 M0-1, who were either unsuitable for surgical intervention or declined surgery, were considered eligible. Tumors were required to be located within 15 cm of the anal verge, with a lumen diameter of at least 2 cm, and could not involve more than two-thirds of the rectal circumference. The 1-year local progression-free survival (PFS) was 64 % and the 12-month OS was 82 % [35]. It should be noted that HDR-BT dose in the HERBERT-I trial was prescribed to a depth of up to 20 mm, which resulted in very high doses to the tumor surface (calculated EQD2 to 150 Gy) that could explain the high incidence of grade 3 rectitis/proctitis (40 %) trial. In contrast, in the here proposed ACO/ARO/AIO-22 trial HDR-BT dose will be prescribed at the radial margin of the tumor, with a maximum prescription depth of 10 mm using strict dose constraints, and, hence, the higher dose of 8 Gy per fraction is expected to be well-tolerated.

A time interval of 2 to 6 weeks between the end of EBRT and HDR-BT has been used in several studies [20,37,46]. In the OPERA trial, a 2–3-week gap was implemented between EBRT and CXB. While a shorter interval could reduce the risk of tumor repopulation, it is often challenging in clinical practice due to the potential onset of acute proctitis or colitis following EBRT. As such, longer waiting interval between EBRT and brachytherapy is meaningful for patient comfort but also in patients with large tumor size to allow for tumor shrinkage. As such, we decided for a 6.5-week interval between EBRT and start of brachytherapy [21].

Altogether, the concept of combining EBRT with endorectal HDR-BT to be tested as part of the ACO/ARO/AIO-22 prospective trial constitutes an attractive option in elderly/frail patients with rectal cancer who are unsuitable for or refuse radical surgery, as it can potentially provide long-term local control, prevent disease-related morbidity, improve QoL and even lead to cCR with complete cure in selected cases.

## Funding

The trial is sponsored by the German Cancer Aid (Deutsche Krebshilfe, Protocol number: 70115482; Principal Investigator (PI): Emmanouil Fokas]. The sponsor will take care of the financing/funding of the study, according to written agreements between the sponsor and the coordinating investigator, the local principal investigators (or their institutions), the contract research organization (CRO) and the source(s) of funding.

## Declaration of Competing Interest

The authors declare that they have no known competing financial interests or personal relationships that could have appeared to influence the work reported in this paper.
