# Supplementary material for: ACO/ARO/AIO-22 – External beam radiotherapy combined with endorectal high-dose-rate brachytherapy in elderly and frail patients with rectal cancer: A prospective multicentre trial of the German Rectal Cancer Study Group
Source: Clin Transl Radiat Oncol. 2025 Apr 13;53:100958. doi: 10.1016/j.ctro.2025.100958 (PMC12020863; doi:10.1016/j.ctro.2025.100958)
Supplement: Supplementary Data 1 [file mmc1.docx]

# Appendix

ACO/ARO/AIO-22 - External beam radiotherapy combined with endorectal high-dose-rate brachytherapy in elderly and frail patients with rectal cancer: A prospective multicentre trial of the German Rectal Cancer Study Group

# Appendix Table 1. G8 geriatric assessment tool in elderly patients established by the siog

| **G8 geriatric tool Items** | **Answers and Score** |
| --- | --- |
| Has food intake declined over the past 3 months due to loss of appetite, digestive problems, chewing, or swallowing difficulties? | 0 = severe decrease in food intake |
|  | 1 = moderate decrease in food intake |
|  | 2 = no decrease in food intake |
| Weight loss during the last 3 months? | 0 = weight loss >3 kg |
|  | 1 = does not know |
|  | 2 = weight loss between 1 and 3 kg |
|  | 3 = no weight loss |
| Mobility? | 0 = bed or chair bound |
|  | 1 = able to get out of bed/chair but does not go out |
|  | 2 = goes out |
| Neuropsychological problems? | 0 = severe dementia or depression |
|  | 1 = mild dementia |
|  | 2 = no psychological problems |
| BMI? (weight in kg)/(height in m^2^) | 0 = BMI <19 |
|  | 1 = BMI 19 to <21 |
|  | 2 = BMI 21 to <23 |
|  | 3 = BMI ≥23 |
| Takes more than three prescription drugs per day? | 0 = yes |
|  | 1 = no |
| In comparison with other people of the same age, how does the patient consider his/her health status? | 0.0 = not as good |
|  | 0.5 = does not know |
|  | 1.0 = as good |
|  | 2.0 = better |
| Age | 0: >85 |
|  | 1: 80–85 |
|  | 2: <80 |
| **Total score**  **A score of ≤ 14 is defined as an impaired G8 tool score(frail patient)* | **0–17** |

**Reference**

van Walree IC, Scheepers E, van Huis-Tanja L, et al: A systematic review on the association of the G8 with geriatric assessment, prognosis and course of treatment in older patients with cancer. J Geriatr Oncol 10:847-858, 2019

# Appendix Table 2. American Society of Anesthesiologists Physical Status (ASA PS) classification

ASA Physical Status Classifications and Examples

| **ASA PS Classification** | **Definition** | **Examples** |
| --- | --- | --- |
| ASA 1 | A normal healthy patient | Healthy, nonsmoking, no or minimal alcohol use |
| ASA 2 | A patient with mild systemic disease | Mild diseases only without substantive functional limitations. Examples include (but not limited to): current smoker, social alcohol drinker, pregnancy, obesity (30 < BMI < 40), well-controlled DM/HTN, mild lung disease |
| ASA 3 | A patient with severe systemic disease | Substantive functional limitations; one or more moderate to severe diseases. Examples include (but not limited to): poorly controlled DM or HTN, COPD, morbid obesity (BMI ≥40 kg/mg^2^), active hepatitis, alcohol dependence or abuse, implanted pacemaker, moderate reduction of ejection fraction, ESRD undergoing regularly scheduled dialysis, premature infant PCA <60 wk, history (>3 mo) of MI, CVA, TIA or CAD/stents |
| ASA 4 | A patient with severe systemic disease that is a constant threat to life | Examples include (but not limited to): recent (<3 mo) MI, CVA, TIA or CAD/stents; ongoing cardiac ischemia or severe valve dysfunction; severe reduction of ejection fraction; sepsis; DIC; ARD; or ESRD not undergoing regularly scheduled dialysis |
| ASA 5 | A moribund patient who is not expected to survive without the operation | Examples include (but not limited to): ruptured abdominal/thoracic aneurysm, massive trauma, intracranial bleed with mass effect, ischemic bowel in the face of significant cardiac pathology or multiple organ/system dysfunction |
| ASA 6 | A declared brain-dead patient whose organs are being removed for donor purposes |  |
| Abbreviations: ARD, acid reflux disease; ASA, American Society of Anesthesiologists; BMI, body mass index; CAD, coronary artery disease; COPD, chronic obstructive pulmonary disease; CVA, cerebral vascular accident; DIC, disseminated intravascular coagulation; DM, diabetes mellitus, ESRD, end-stage renal disease; HTN, hypertension; MI, myocardial infarction; PCA, postconceptual age; PS, physical status; TIA, transient ischemic attack | | |

# SOP EXTERNAL BEAM RADIOTHERAPY

## 1. General Aspects

External beam radiotherapy (EBRT) should be given to the patients as either intensity modulated radiotherapy (IMRT) or volumetric modulated arc therapy (VMAT) and should be continued for the entire course of treatment. IMRT or VMAT technique is now preferred as they result in significantly lower radiation exposure to the organs at risk.^1^ Megavoltage equipment equal to or greater than 6 MeV photons is mandatory. A multi-leaf collimator is required to allow customized blocking and intensity modulation. The radiotherapy treatment volumes are chosen by the treating radiation oncologist on the basis of individual clinical factors (e.g., patient anatomy).

## 2. Immobilization and Planning CT Scan

The use of specific positioning devices to treat the abdominopelvic region is mandatory. We recommend treating the patient in prone position, if prone position is not feasible, the patient can be treated supine. Measures should be taken to reduce the exposure of the small bowel and, in the case of male patients, of the testes, e.g., by using a (double) belly board device or open table top device. A treatment planning CT scan is mandatory for defining the target volume, CT scan thickness should preferably be 3 mm but not exceed 5 mm. The treatment planning CT scan must be acquired with the patient in the same position and using the same immobilization device as for treatment. All tissues receiving any irradiation must be included in the CT scan.

## 3. Target Volumes of external beam radiotherapy

On all appropriate CT slices, the gross target volume (GTV) and the clinical target volume (CTV) should be outlined. For the GTV, the primary lesion and suspect lymph nodes (defined on pre-treatment examinations including pelvic MRI) should be delineated separately. On all appropriate CT slices, the gross target volume (GTV) and the clinical target volume (CTV) should be outlined. For the GTV, the primary lesion and suspect lymph nodes (defined on pre-treatment examinations including CT/MRI) should be delineated separately.

Typical target volumes have incorporated the primary tumor and any gross disease, with elective irradiation of the whole mesorectum, the presacral and internal iliac node with cranial limits around the level of the sacral promontory all receiving the same dose fractionation. An analysis of the patterns of failure of patients in the Dutch TME trial demonstrated that nearly all of the local recurrences were found below the level of the S2/3 interspace^2,3^.

In the past, target volume delineation was characterized by large inhomogeneity among radiation oncologists that led to systematic errors with a standard deviation up to 1 cm ^2,4^. Following the advent of conformal RT recommendations on contouring from different societies have been published ^5-8^, of which two have been extensively used: those published by Roels et al. from Europe and those provided by RTOG from US ^6,7^. All these guidelines provide boundaries, atlas and recommendations for pelvic CTV's delineation. With the aim to homogenize delineation in rectal cancer, leading experts from the Radiotherapy Cancer Societies (ESTRO, ASTRO, The Royal Australian and New Zeland College of Radiologists and EORTC) and authors of one of the available published guidelines cooperated in a project to review the current published guidelines and to propose shared rectal cancer delineation guidelines^9^. These guidelines by Valentini et al. published in the Green Journal in 2016 were intended only for elective CTV. The consensus process was based on review of the current literature contouring guidelines, review of site of local recurrences, review of the radiological and surgical anatomy, and delineation of 7 real clinical cases at different tumor stages. The guidelines for rectal CTV delineation aimed to increase the conformity in volumes nomenclature among different radiation oncologists, and should be consulted by the local radiation oncologist for the radiotherapy of patients within the trial ^9^.

However, in the guidelines by Valentini et al. described above, elective node irradiation volumes were still recommended even in patients with early rectal cancer i.e. stage cT1-3bN0. Notably, the risk of pelvic lymph node involvement or distal mesorectal nodal involvement is very low in these patients ^6,10^. It is unlikely that such large elective irradiation volumes are indicated in early rectal cancers especially as most will be clinically node negative and microscopic disease will be predominantly confined to the mesorectum. It is therefore reasonable to reduce the target volume to the peritumoral region of the primary tumour and the mesorectum^11^.

This concept of mesorectal CTV has been adopted in the STAR-TREC trial ^12^ to significantly reduce the irradiation volume in the -caudal direction and to avoid the obturator nodes anteriorly, thus decreasing treatment-related toxicity without compromising oncological outcomes. The STAR-TREC trial explores whether primary short-course radiotherapy (SCRT) or CRT followed by a two-stage response assessment with selective use of local excision, is a safe alternative to TME surgery (ClinicalTrials.gov Identifier: NCT02945566). Patients with small cT1-3bN0M0 tumors are randomized between three arms: standard TME, organ preservation (OP) with SCRT or with CRT. Patients in the organ preservations arms with a complete clinical response (cCR) enter active surveillance without further treatment. For patients with a good partial response the residual disease will be locally excised, while in poor responders a TME resection is advocated. As such, in patients with stage cT1-3bN0 rectal cancer, CTV has been tailored to include the mesorectum and pre-sacral lymph nodes at the level of the tumor, two centimetres below and cranially up to the S2-3 interspace level, whereas the lateral lymph nodes and the nodes along the superior rectal artery are excluded^12^. As a result, the dose to the bowel, bladder, anal sphincter and the neurovascular plexus in the lower pelvis is substantially decreased, especially when combined with modern irradiation techniques, such as IMRT/VMAT. These lower doses are expected to substantially decrease acute and late toxicity with beneficial functional outcomes for patients with cT1-3bN0 rectal cancer. Illustration of the CTV definition between the international consensus recommendations by Valentini et al. and the STAR-TREC recommendations by Peters et al. for patients with cT1-3bN0 rectal cancer is shown below in **Table 1.**

| **Table 1.** Comparison of the CTV definition between the International consensus recommendations and the STAR-TREC recommendations in patients with **cT1-3bN0 rectal cancer** | | |
| --- | --- | --- |
| **Anatomical areas** | **International consensus recommendations**^9^ | **STAR-TREC protocol recommendations**^12^ |
| Mesorectum | + | +, <S2-3 |
| Presacral Nodes, pelvic | + | +, <S2-3 |
| LLN post. = internal iliac LN | + | – |
| LLN ant. = obturator LN | – | – |
| Sphincter Complex | – | – |
| External Iliac Nodes | – | – |
| Ischiorectal Fossa | – | – |
| Inguinal Nodes | – | – |
| Presacral Nodes, abdominal | – | – |

***Abbreviations:*** *LLN, lateral lymph nodes;*

Based on the above, and considering that the risk of radiotherapy-related side effects is higher in elderly/frail patients with rectal cancer compared to younger and fit patients, **we recommend** **a disease stage-based approach for the contouring of CTV in the present ACO/ARO/AIO-22 trial** as summarized below:

**- *cT1-3bN0:*** CTV contouring should be performed based on the STAR-TREC protocol recommendations by Peters et al ^12^.

***- cT3c-T4N0-N2 or cTany N1-2:*** CTV contouring should be performed based on the ESTRO/ASTRO/RANZCR/EORTC International consensus recommendations by Valentini et al ^9^.

## 4. Target volume contouring in patients with cT1-3bN0 rectal cancer, based on the STAR-TREC protocol recommendations

Target volume contouring in patients with cT1-3bN0 rectal cancer, based on the STAR-TREC protocol recommendations is summarized in **Table 2**. The CTV includes the mesorectum considered at risk for LN involvement and the pre-sacral LN at the same level. MRI should be used to aid. Since all tumours should be amenable for local excision, proximal anterior located tumours (above peritoneal fold) and distal tumours with extension in the anal canal are not eligible for inclusion in the STAR-TREC trial. The delineation guidelines are, therefore, not applicable for these locations. Required PTV margins are highly dependable of local techniques, such as positioning and position verification. A strict instruction is, therefore, not in place. In that case, a PTV margin of at least 1.5 cm anteriorly and 1 cm in all other directions should be used. If less than daily online position verification is used or, for example, the patient is in prone position without belly board, appropriate extra PTV margin should be added.

| **Table 2.** Target volume contouring in patients with **cT1-3bN0** rectal cancer, based on the STAR-TREC protocol recommendations by Peters et al ^12^ | |
| --- | --- |
| **GTV** | All macroscopic tumor is delineated on each slice. |
| **CTV** | **On each slice, the mesorectal fascia is delineated circumferentially:**  ***Superior limit:***   - Is defined as the S2/S3 interspace (determined on the sagittal or scout view on the planning system). - minimum of 2 cm is required from the superior limit of the GTV to the CTV. (In superiorly placed tumors, this may require an extension of the CTV above the S2/3 interspace to achieve the 2 cm margin.)   ***Inferior limit:***   - Is defined as 2 cm inferior to the inferior limit of the GTV. - In low tumors, where a 2 cm margin extends below the end of the mesorectum and into the anal canal, this margin is reduced to 1cm. (The anal canal is delineated if the CTV extends below the mesorectum)   ***Anterior limit:***   - The mesorectal fascia is contoured. - If the mesorectal fascia disappears anteriorly, the anterior border is the anterior rectal wall. - For cranial slices with no visible rectum, the anterior border is defined by the contour used for the last cranial slice with visible rectum.   ***Posterior limit:***   - Is defined as the anterior margin of the sacrum or coccyx, or the inner border of the puborectalis muscle in caudal slices.   ***Lateral limit:***   - The mesorectal fascia is contoured. - High pelvis - If the mesorectal fascia disappears laterally, the inner border of the pyriformis muscle is contoured - Mid pelvis - The mesorectal fascia is contoured. - Low pelvis - The inner border of the puborectalis muscle as it converges to form the anorectal ring. |
| **PTV** | - CTV with a 1cm isotropic margin applied superiorly, inferiorly, posteriorly and laterally, and a 1.5cm isotropic margin applied anteriorly. - If there is no daily on-treatment image-guidance, an additional isotropic margin (according to local policies) for set-up error is to be added. |

## 5. International consensus recommendation for CTV delineation in patients with cT3c-T4N0-N2 or cTany N1-2 rectal cancer

### 5.1 Definition of anatomical borders for CTV delineation in patients with cT3c-T4N0-N2 or cTany N1-2 rectal cancer

In the International consensus recommendation guidelines by Valentini et al^9^. several subsites were described as follows: Presacral nodes (PN), Mesorectum (M), Lateral lymph nodes (LLN), External iliac nodes (EIN), Ischio-rectal fossa (IRF), Sphincter complex (SC), Inguinal Nodes (IN). The borders of these subsites are defined in detail below in **Table 3.**

| Table 3. Definition of anatomical borders for CTV delineation in patients with cT3c-T4N0-N2 or cTany N1-2 rectal cancer, based on the International consensus recommendations by Valentini et al. | | |
| --- | --- | --- |
|  | **Subsites** | **Borders** |
| PS | Abdominal | *Cranial:* bifurcation of the aorta in common iliac arteries or 5 mm above the most cranial positive lymph-node |
|  |  | *Caudal:* sacral promontory |
|  |  | *Anterior:* 1 cm ventral to the lumbar vertebrae |
|  |  | *Posterior:* anterior wall of the lumbar vertebrae |
|  |  | *Medial:* – |
|  |  | *Lateral:* lateral surface of the common iliac vessels |
|  | Pelvic | *Cranial:* bifurcation of the common iliac arteries into the external and internal iliac artery/sacral promontory |
|  |  | *Caudal:* caudal border of the M |
|  |  | *Anterior:* 1 cm ventral to the lumbar vertebrae |
|  |  | *Posterior:* anterior wall of the sacral vertebrae |
|  |  | *Medial:* – |
|  |  | *Lateral:* sacroiliac joints |
| M |  | *Cranial:* bifurcation of the IMA in SA and SRA |
|  |  | *Caudal:* insertion of the levator ani muscle into the external sphincter muscles (disappearing of the mesorectal fat around the rectum) |
|  |  | *Anterior* *Superior:* 7 mm beyond SRA excluding bowel structures *Mid/inferior:* mesorectal fascia, posterior border of the anterior pelvic organs |
|  |  | *Posterior:* Anterior surface of the sacrum and coccyx to the level of IRF (including the medial part of the PS) |
|  |  | *Medial:* – |
|  |  | *Lateral:* *Upper/mid:* Mesorectal fascia if visible or medial border of the LLN and EIN *Lower:* medial edge levator ani muscle |
| LLN | Posterior  (ex internal iliac nodes) | *Cranial:* Bifurcation of common iliac artery into internal and external iliac arteries |
|  |  | *Caudal:* insertion of the levator ani muscle into the external sphincter muscles (pelvic floor) |
|  |  | *Anterior* *Upper pelvis:* 7 mm around the vessel. *Mid pelvis:* a virtual coronal plane crossing the anterior wall of the ureters when they join the bladder and the posterior aspect of the external iliac vessels cranially *Inferior pelvis: po*sterior limit of the obturator fossa |
|  |  | *Posterior:* Lateral edge of the sacro-iliac joint |
|  |  | *Medial:* *Upper:* Above the M add 7 mm around the vessel, excluding normal anatomic structures *Mid/lower:* Mesorectal fascia, pelvic organs |
|  |  | *Lateral* *Upper:* iliopsoas, pelvic bones *Mid-lower:* medial edge of the pelvic wall muscles (pyriform and internal obturator muscles) |
|  | Anterior  (ex obturator nodes) | *Anterior Mid pelvis:* posterior wall of the EIN *Low pelvis* (when external iliac vessels leave the pelvis): anterior surface of obturator artery |
| EIN |  | *Cranial:* bifurcation of common iliac artery into internal and external iliac arteries |
|  |  | *Caudal:* where the deep circumflex vein crosses the external iliac artery. Alternatively (if difficult detection on CT images) between the acetabulum roof and the superior pubic rami |
|  |  | *Anterior:* 0.7 cm anterior to the vessels. 1.5 cm antero-laterally along the iliopsoas muscle to include the antero-lateral nodes |
|  |  | *Posterior:* posterior border of the external iliac vein |
|  |  | *Medial:* 7 mm medial to the vessel, excluding pelvic organs |
|  |  | *Lateral:* the iliopsoas muscle |
| IN |  | *Cranial:* where the deep circumflex vein crosses the external iliac artery. Alternatively (if difficult detection on CT images) between the acetabulum roof and the superior pubic rami |
|  |  | *Caudal:* where the great saphenous vein enters the femoral vein |
|  |  | *Anterior:* at least 20 mm margin around inguinal vessels including any visible lymph nodes or lymphoceles |
|  |  | *Posterior:* the femoral triangle formed by iliopsoas, pectineus and abductor longus muscles |
|  |  | *Medial:* 10-20 mm margin around the femoral vessels including any visible lymph nodes or lymphoceles |
|  |  | *Lateral:* medial edge of the sartorius or iliopsoas muscles |
| IRF |  | *Cranial:* where the inferior pudendal artery leaves the pelvis (ischial tuberosity, internal obturator muscle, gluteus maximus muscle) |
|  |  | *Caudal:* oblique plane joining the inferior level of SC and the ischial tuberosity. |
|  |  | *Posterior:* *Mid-superior:* major gluteus muscle *Inferior:* a virtual line tangent to the posterior level of the sphincter |
|  |  | *Medial:* levator ani muscle |
|  |  | *Lateral:* ischial tuberosity, internal obturator muscle, Gluteus maximus muscle |
| SC |  | From the anal-rectal junction. Around the sphincter |

***Abbreviations:*** *PS: Presacral space, M: Mesorectum, LLN: Lateral Lymph Nodes, EIN: External Iliac Nodes, IN: Inguinal Nodes, IRF: Ischio-Rectal Fossae, SC: Sphincter Complex, IMA:* [*inferior mesenteric artery*](https://www.sciencedirect.com/topics/medicine-and-dentistry/inferior-mesenteric-artery)*, SA:* [*sigmoid*](https://www.sciencedirect.com/topics/medicine-and-dentistry/sigmoid) *artery, SRA: superior* [*rectal artery*](https://www.sciencedirect.com/topics/medicine-and-dentistry/rectal-artery)*.*

### 5.2 Delineation of the CTV according to tumor TNM stage and location in patients with cT3c-T4N0-N2 or cTany N1-2 rectal cancer

An example of CTV contouring based on the international consensus panel ^9^ is shown in **Figure 2** but we refer to the original consensus recommendation atlas for further examples. The guidelines also provided suggestions for the modulation of the elective CTV according to T and N stage are summarized in **Table 4** and described in detail below:

1. Always include the mesorectum, the pelvic presacral space and the posterior lateral nodes in the CTV. Abdominal presacral nodes should be included only when positive nodes are identified in this area.
2. The posterior lateral nodes i.e., the internal iliac nodes should always be included in the CTV. In case of cT4 tumors, N2 lymph node involvement or positive posterior lateral nodes, the anterior lateral nodes, i.e., the obturator nodes must be included in the CTV.
3. The cranial border of the CTV can be modulated, as previously described ^3,13^. The superior border can be limited at the level of the start of the mesorectum (bifurcation of the inferior mesenteric artery in sigmoid artery and superior rectal artery) in case of cT3 tumors not reaching the mesorectal fascia (MRF-) and with negative mesorectal nodes; in all other cases (MRF+/cT4 and/or positive lymph nodes), the superior limit should be positioned at the bifurcation of the common iliac in external and internal vessels; in case of higher tumor the upper limit should be at least 1 cm above the most cranial part of the primary lesion. In the case of positive abdominal nodes at PS the cranial limit should be increased to the aorta bifurcation or 0.5 cm above the most cranial positive node.
4. External iliac nodes must be included only when the tumor is extensively invading the anterior organs (cT4) or in case of positive nodes in the anterior lateral nodes area.
5. Inguinal nodes must be included when they are positive, when tumor extends to the anal canal below the dentate line, in case of infiltration of the anal sphincter or lower third of the vagina.
6. Ischiorectal fossae and sphincter complex must be encompassed in the CTV when one or both are infiltrated by the tumor.

| Table 4. CTV contouring for cT3c-T4N0-N2 or cTany N1-2 rectal cancer depending on the tumor stage and location in rectal cancer, based on the International Consensus recommendations by Valentini et al. | | | | | | | | | |
| --- | --- | --- | --- | --- | --- | --- | --- | --- | --- |
|  | **M** | **PS** | | **LLN** | | **EIN** | **IRF** | **IN** | **SC** |
|  |  | **Pelvic** | **Abdominal** | **Post** | **Ant** |  |  |  |  |
| cT3 | + | + | When LN+ | + | + (in case of numerous mesorectum nodes (N2) |  |  |  |  |
| cT4  (anterior pelvic organ) | + | + | When LN+ | + | + | + |  | + (in case of infiltration of inferior third of vagina) |  |
| cT4  (anal sphincter) | + | + | When LN+ | + | + | + | + (when direct tumor infiltration of IRF or external anal sphincter) | + | + |
| cT3 with extra mesorectal node | + | + | When LN+ | + | + | + |  |  |  |

***Abbreviations:*** *M: mesorectum, PS: Presacral Space LLN: Lateral Lymph Nodes, EIN: External iliac Nodes, IRF: Ischiorectal Fossa,IN: Inguinal Nodes, SC: Sphincter Complex.*

### 5.3 PTV delineation in patients with cT3c-T4N0-N2 or cTany N1-2 rectal cancer

On all appropriate CT slices, the GTV and the CTV are outlined to generate the final planning target volume (PTV). The PTV represents an additional margin around the CTV to compensate for the variability of treatment set up and internal organ motion. A minimum margin of at least 0.5 cm around the CTV is required in all directions to define a respective PTV. Additional margins may be required based upon clinical judgment, especially in the case of suspected or diagnosed lymph node metastases. Importantly, the exact PTV margins used will depend on the in-house radiotherapy protocol for set-up errors but the minimum recommended CTV-PTV margins should be 0.5 cm-1cm to incorporate outlining uncertainties. Examples of the CTV contours from the international consensus panel ^9^ are shown in **Figure 2** below and can be used for the radiotherapy within this trial.

## 6. Organs at risk (OAR)

The bowel bag is usually outlined while the delineation of the bowel loops is added only in case they are placed close to the CTV or fixed in the pelvis. The small bowel is contoured from the Douglas pouch up to at least 1 cm above the cranial PTV margin. The whole bladder is outlined, whereas planning CT scan and radiotherapy are performed with full bladder. The rectum is contoured from the ischial tuberosities up to the flexion at the junction with the sigmoid. Furthermore, the anus and the genitalia should be contoured. A contouring atlas has been previously published by the RTOG and can be used for the delineation of OAR ^14^.

## 7. Dose Prescription and Dose Specification of external beam radiotherapy

External beam radiotherapy to the PTV (primary tumor and pelvic lymph nodes) will be given in 13 fractions of 3 Gy per day to a total dose of 39 Gy over 2.5 weeks. For IMRT and VMAT, the digital dose distribution should be defined according to the ICRU report 83 at the D_50%_. Additionally, for both treatment arms:

- - The isodose curve representing the 95% of the prescribed dose must encompass the entire PTV.
  - No part of the PTV should receive ≥ 107% of the prescribed dose.
  - Replacement of the PTV point dosages D_min_ and D_max_ by the volume-based dosages D_98%_ and D_2%_ of the DVHs should be considered for all techniques
  - Less than 1% or 1cc of the tissue outside the PTV should receive ≥ 107% of the prescribed dose to the PTV and Boost.
  - The constraints to the organs at risk (OAR) should be considered during preparation of the radiotherapy plans. Albeit there are enough data on the constraints to the OAR after the classical long-course (chemo-)radiotherapy, there are no established OAR constraints for the EBRT regimen of 13 x 3 Gy to 39 Gy applied in the present ACO/ARO/AIO-22 trial. For the ACO/ARO/AIO-22 trial the choice of the OAR constraints is left to the discretion of the local radiation oncologist. We have, nevertheless, recalculated the OAR constraints for EQD2 (Gy) with the LQ-model, based on the National rectal cancer intensity-modulated radiotherapy (IMRT) guidance recommendations of the Royal College of Radiologists UK^15^.

| **Organ at risk** | **Constraints to the OAR for EBRT with 13 x 3 Gy to 39 Gy** |
| --- | --- |
| Bowel cavity | D400cc <19 Gy |
|  | D250cc <28 Gy |
|  | D200cc <40 Gy |
| Bladder | D50% <33 Gy |
|  | D35% <38 Gy |
|  | D5% <47 Gy |
| Femoral heads | D50% <28 Gy |
|  | D35% <38 Gy |
|  | D5% <47 Gy |
| Genitalia | D50% <19 Gy |
|  | D35% <28 Gy |
|  | D5% <38 Gy |

## 8. Documentation Requirements and Portal Films

Portal image of each field or orthogonal images that localize the isocenter placement must be obtained on the first day of therapy. Isodose plans, DVHs of the target volumes and critical normal structures are mandatory for planning. Weekly positioning controls of the patients are required. Image guided radiotherapy (IGRT) with daily positioning control is allowed, but not mandatory.

**References**

1. Guerrero Urbano MT, Henrys AJ, Adams EJ, et al. Intensity-modulated radiotherapy in patients with locally advanced rectal cancer reduces volume of bowel treated to high dose levels. *Int J Radiat Oncol Biol Phys.* 2006;65(3):907-916.

2. Nijkamp J, de Haas-Kock DF, Beukema JC, et al. Target volume delineation variation in radiotherapy for early stage rectal cancer in the Netherlands. *Radiother Oncol.* 2012;102(1):14-21.

3. Nijkamp J, Kusters M, Beets-Tan RG, et al. Three-dimensional analysis of recurrence patterns in rectal cancer: the cranial border in hypofractionated preoperative radiotherapy can be lowered. *Int J Radiat Oncol Biol Phys.* 2011;80(1):103-110.

4. Fuller CD, Nijkamp J, Duppen JC, et al. Prospective randomized double-blind pilot study of site-specific consensus atlas implementation for rectal cancer target volume delineation in the cooperative group setting. *Int J Radiat Oncol Biol Phys.* 2011;79(2):481-489.

5. Ng M, Leong T, Chander S, et al. Australasian Gastrointestinal Trials Group (AGITG) contouring atlas and planning guidelines for intensity-modulated radiotherapy in anal cancer. *Int J Radiat Oncol Biol Phys.* 2012;83(5):1455-1462.

6. Roels S, Duthoy W, Haustermans K, et al. Definition and delineation of the clinical target volume for rectal cancer. *Int J Radiat Oncol Biol Phys.* 2006;65(4):1129-1142.

7. Myerson RJ, Garofalo MC, El Naqa I, et al. Elective clinical target volumes for conformal therapy in anorectal cancer: a radiation therapy oncology group consensus panel contouring atlas. *Int J Radiat Oncol Biol Phys.* 2009;74(3):824-830.

8. Arcangeli S, Valentini V, Nori SL, Fares C, Dinapoli N, Gambacorta MA. Underlying anatomy for CTV contouring and lymphatic drainage in rectal cancer radiation therapy. *Rays.* 2003;28(3):331-336.

9. Valentini V, Gambacorta MA, Barbaro B, et al. International consensus guidelines on Clinical Target Volume delineation in rectal cancer. *Radiother Oncol.* 2016;120(2):195-201.

10. Leibold T, Shia J, Ruo L, et al. Prognostic implications of the distribution of lymph node metastases in rectal cancer after neoadjuvant chemoradiotherapy. *J Clin Oncol.* 2008;26(13):2106-2111.

11. Marijnen CA. Organ preservation in rectal cancer: have all questions been answered? *Lancet Oncol.* 2015;16(1):e13-22.

12. Peters FP, Teo MTW, Appelt AL, et al. Mesorectal radiotherapy for early stage rectal cancer: A novel target volume. *Clin Transl Radiat Oncol.* 2020;21:104-111.

13. Joye I, Haustermans K. Clinical target volume delineation for rectal cancer radiation therapy: time for updated guidelines? *Int J Radiat Oncol Biol Phys.* 2015;91(4):690-691.

14. Gay HA, Barthold HJ, O'Meara E, et al. Pelvic normal tissue contouring guidelines for radiation therapy: a Radiation Therapy Oncology Group consensus panel atlas. *Int J Radiat Oncol Biol Phys.* 2012;83(3):e353-362.

15. UK. NrcIg. https://www.rcr.ac.uk/publication/national-rectal-cancer-intensity-modulated-radiotherapy-imrt-guidance, 2021.

# SOP ENDORECTAL HIGH-DOSE-RATE BRACHYTHERAPY

**General Aspects**

The endorectal HDR brachytherapy (HDR-BT) is administered in three consecutive sessions, each conducted weekly, starting 6.5 weeks after the completion of external beam radiotherapy (EBRT). During the first week of brachytherapy, a diagnostic *rectal* MRI (follow-up MRI) and a rectoscopy are performed once priorly to therapy. The MRI is conducted first, followed promptly by the rectoscopy. The diagnostic pelvic MRI will be conducted to evaluate the therapeutic response achieved thus far. Tumor localization should be documented, including a description of morphological changes and the specific anatomical location according to transverse anatomical reference planes (e.g., affected rectal wall side, tumor length, and circumferential involvement).

Shortly after the follow-up MRI, a diagnostic rectoscopy is performed on the day prior to the first endorectal HDR-BT session. This procedure provides a detailed description of tumor extension, also using transverse anatomical reference planes. Documentation must include the cranial and caudal tumor margins (measured in centimeters from the dentate line), the extent of circumferential involvement (specified as clock positions), and clip marking of the clinically detectable tumor borders (cranial, caudal, and lateral). On the same day, immediately following the rectoscopy, a reference CT scan of the pelvis is performed in the treatment position (supine) in radiotherapy, with thin slices (≤3 mm, whereas 1.5 mm slice thickness is recommended)) to document the topographical anatomy and clip locations within the rectum. This CT serves as fusion imaging for 3D radiation planning during brachytherapy sessions and for verifying anatomical consistency based on rectoscopy and MRI findings. MRI following rectoscopy should be avoided due to the risk of trauma- or inflammation-induced changes caused by the clip placement, which could compromise diagnostic accuracy.

The first endorectal HDR-BT session is conducted the day after rectoscopy with the patient fasting. The treatment is performed without systemic anesthesia or sedation. Local analgesia, typically via rectal instillation of a lidocaine-containing gel, is generally sufficient. Subsequent endorectal HDR-BT sessions are performed on a weekly basis under fasting conditions without repeated rectoscopy or rectal MRI. Preparation for HDR-BT includes bowel-cleansing measures similar to those used for colonoscopy, employing approved preparations beginning the day prior to treatment. Patients are positioned on a specialized brachytherapy table (for example Brachy T-Table, GMmbH, Groß-Gerau, Germany, or equivalent), which facilitates seamless patient transfer and ensures consistent positioning and applicator geometry during CT imaging and HDR-BT.

The use of various applicators from different manufacturers is feasible. The procedural details of brachytherapy application are described below for the flexible multichannel rectal applicator by Elekta (this example refers to an older model, which is no longer available as of January 2025. A successor model is expected to be approved and available from mid-2025 onwards) and the multichannel cylinder by Varian.

The following sections provide an exemplary overview of potential materials and the workflow for brachytherapy. **Figure 1** illustrates an example of CT-based radiation therapy. **Figure 2** demonstrates endoscopic findings in a patient with macroscopic residual tumor with fibrin coating after EBRT, including clip placement and cCR 8 weeks after completion of brachytherapy. **Figures 3–5** showcase the equipment for endorectal brachytherapy as well as the aforementioned applicators. **Figures 6–16** present a step-by-step guide to the possible implementation of a brachytherapy application within the framework of the study, using the Elekta applicator (older model) as an example (additional: fixation of the MCC applicator (by Varian) within the holder).


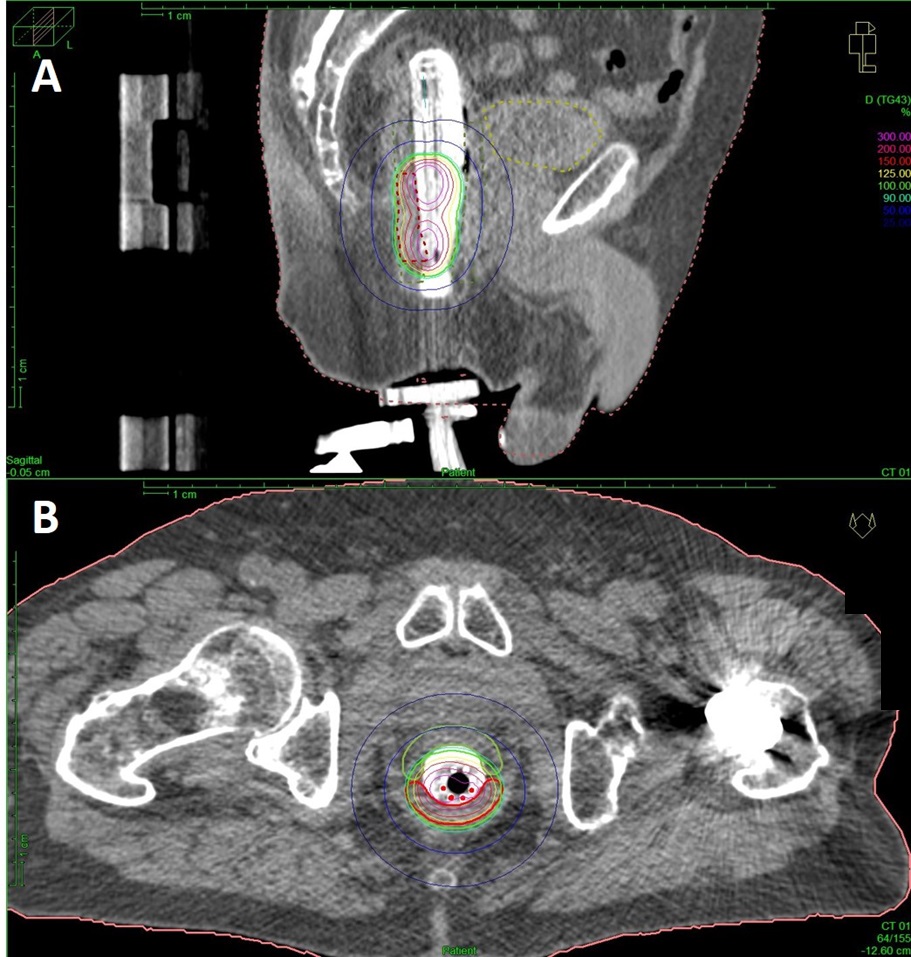


**Figure 1:** CT-Based treatment planning (example). Transverse and sagittal images of a CT-based treatment plan and dose distribution. A semicircular balloon filled with radiopaque contrast agent serves as a spacer to increase the distance between the target volume and the contralateral rectal wall, ensuring stable positioning and proper contact surface. The applicator is additionally secured to the treatment table using a specialized clamp. Isodose Color Code Convention: Green: 100%, Red 150%, Magenta: 300%. The planning target volume (PTV) is also delineated in red.

**
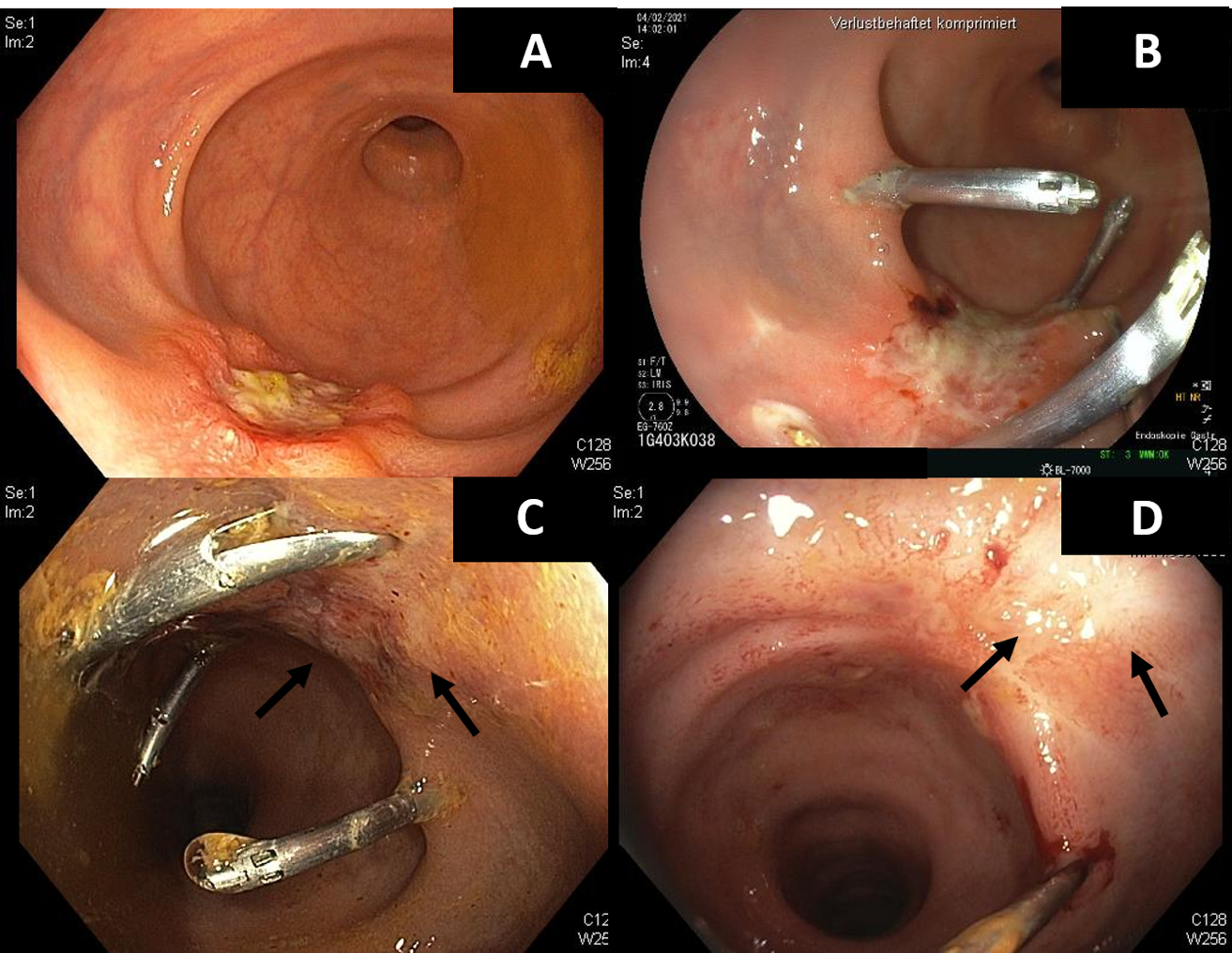
**

**Figure 2:** Endoscopic findings in a patient with cCR after EBRT followed by HDR-BT. (A) Eight weeks after 13 × 3 Gy EBRT, a macroscopic residual tumor with fibrin coating is visible in the distal rectum. The extent of the residual tumor in distal, proximal, and lateral directions is determined using radiopaque markers (clip placement). (B) After two weekly fractions of HDR-BT, the first endoscopy reveals a significant tumor response. (C) Following three fractions of HDR-BT, near cCR is observed, with only small residual ulcerations remaining. (D) Clinical complete remission (cCR) is achieved eight weeks after treatment. The arrows indicate minimal erythema and a residual scar.


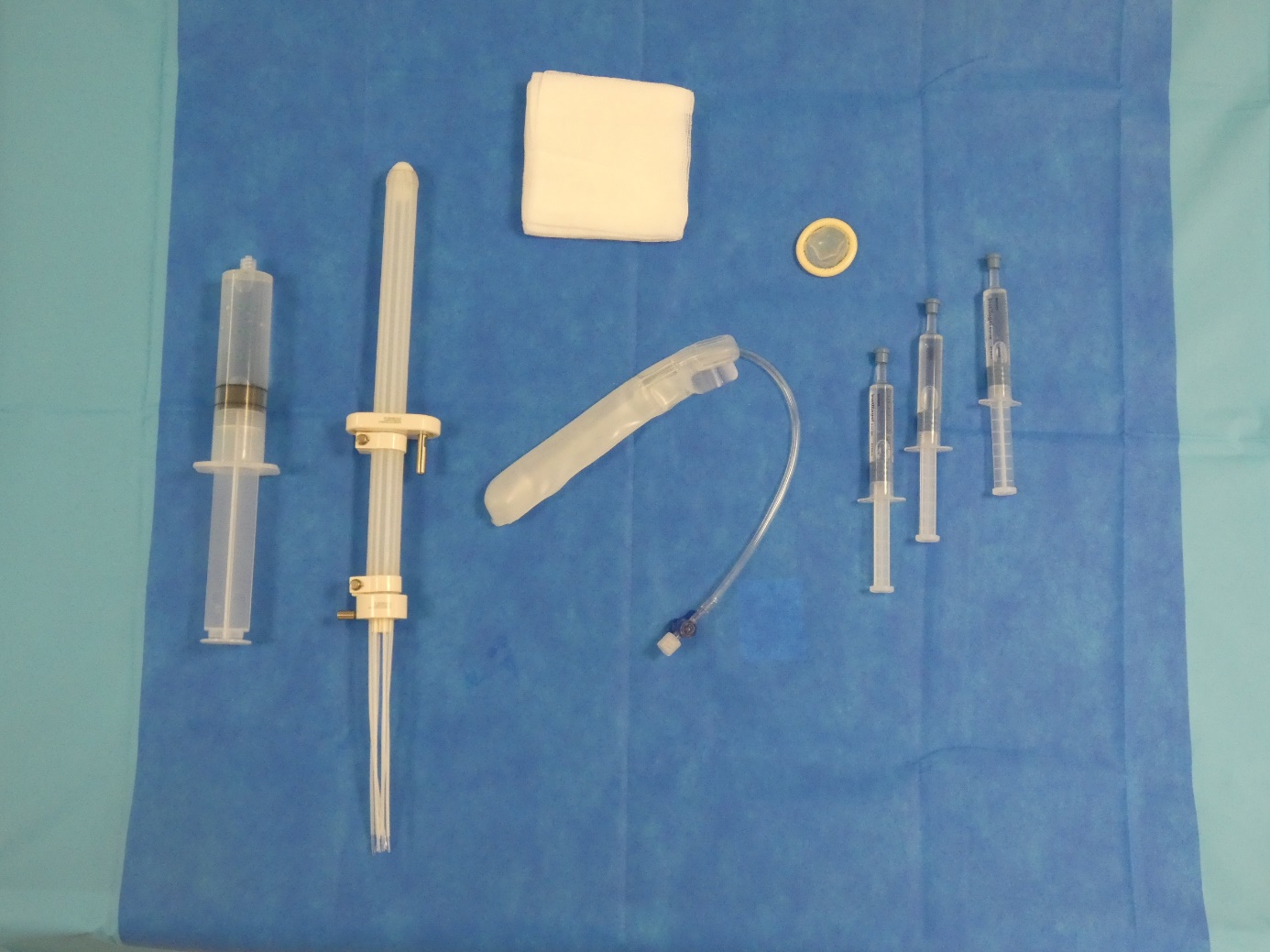


**Figure 3a:** Equipment for endorectal brachytherapy. From left to right:

- Infusomat syringe pump
- Flexible multichannel rectal applicator by Elekta© (older model)
- Single-chamber cover balloon for endorectal ultrasound (brachytherapy balloon)
- Gauze compresses (10 × 10 cm)
- Protective condom for the multichannel rectal applicator
- Instillagel© for endorectal application prior to applicator insertion


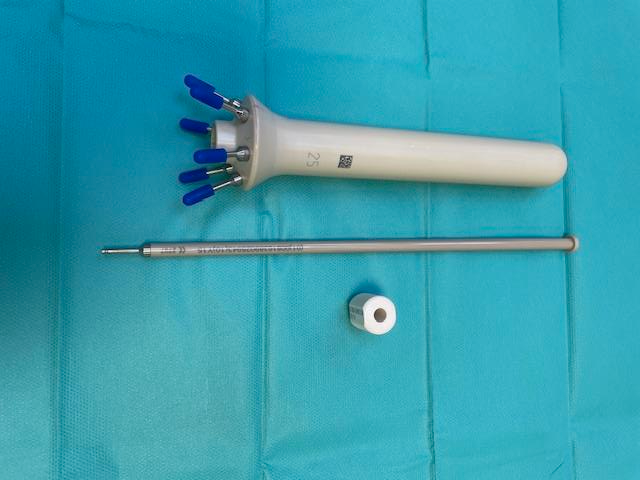


**Figure 3b:** Alternative applicator by Varian©. MCC, Universal Multi-Channel Cylinder. Diameter: 25 mm. Features 7 channels (1 central channel and 6 peripheral channels, numbered). Includes a 250 mm rigid guide tube, clamping segment, and cleaning caps (dark blue).

**
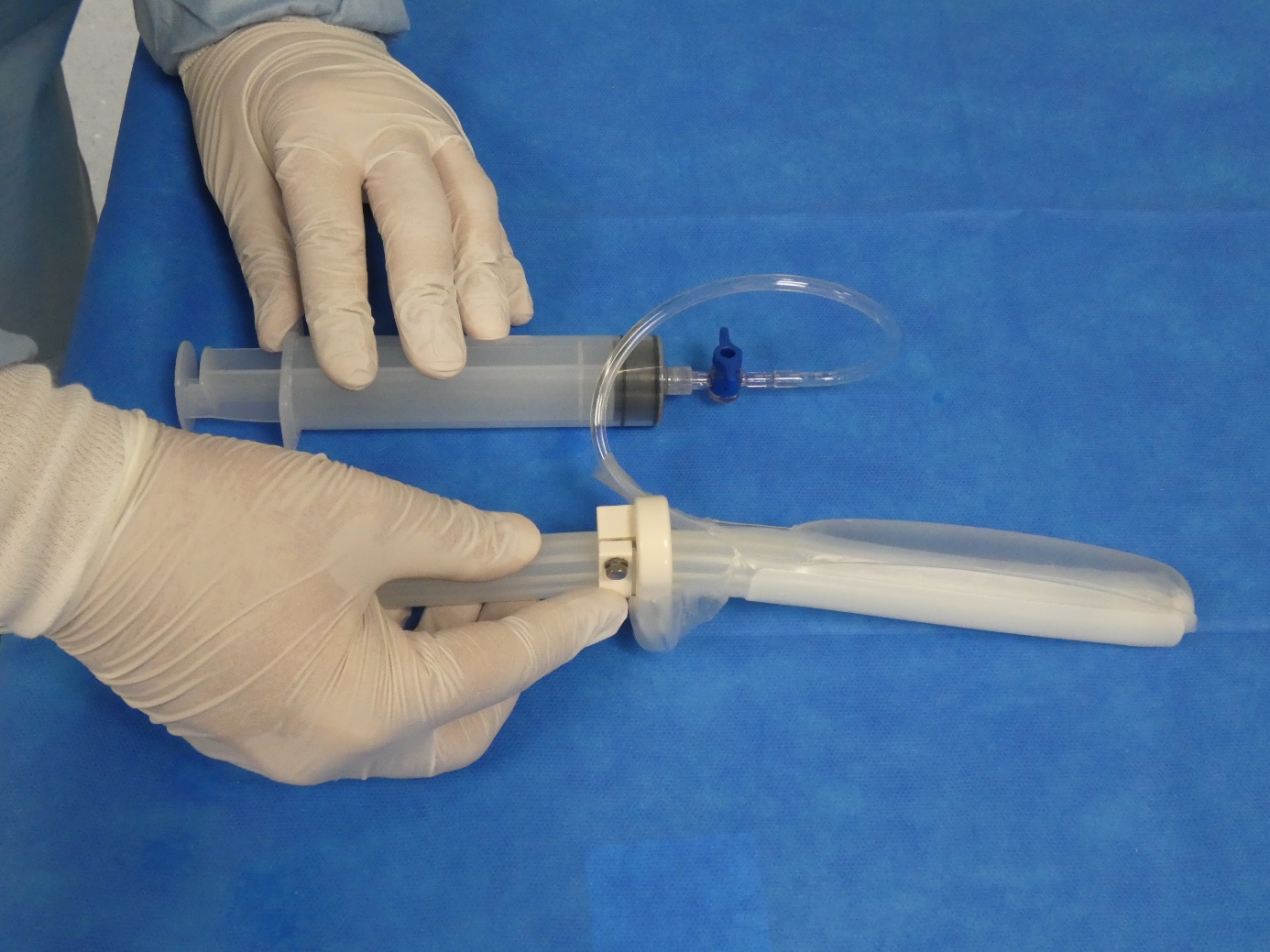
**

**Figure 4a:** Single-Chamber cover balloon for treatment. The single-chamber cover balloon is mounted over the applicator. Chamber filling is performed by injecting saline using the Infusomat syringe. The balloon is filled only after rectal insertion of the applicator and with the patient in the supine position. The injection volume ranges from 20–50 ml of saline, depending on the patient’s tolerance. This illustration provides a preview of the applicator in its final configuration prior to HDR irradiation.


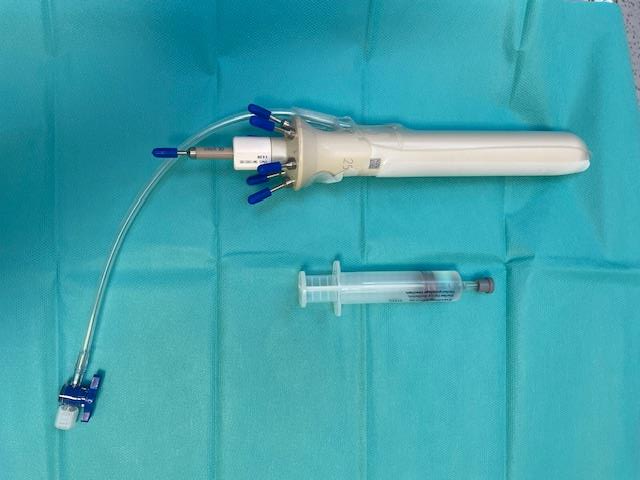


**Figure 4b:** MCC by Varian© with Single-Chamber Cover Balloon. The single-chamber cover balloon is placed over the MCC applicator. The process of sliding the balloon over the MCC is facilitated by the use of a small amount of gel and should be pulled over slowly while continuously pressing out the remaining air at the balloon tip.

**
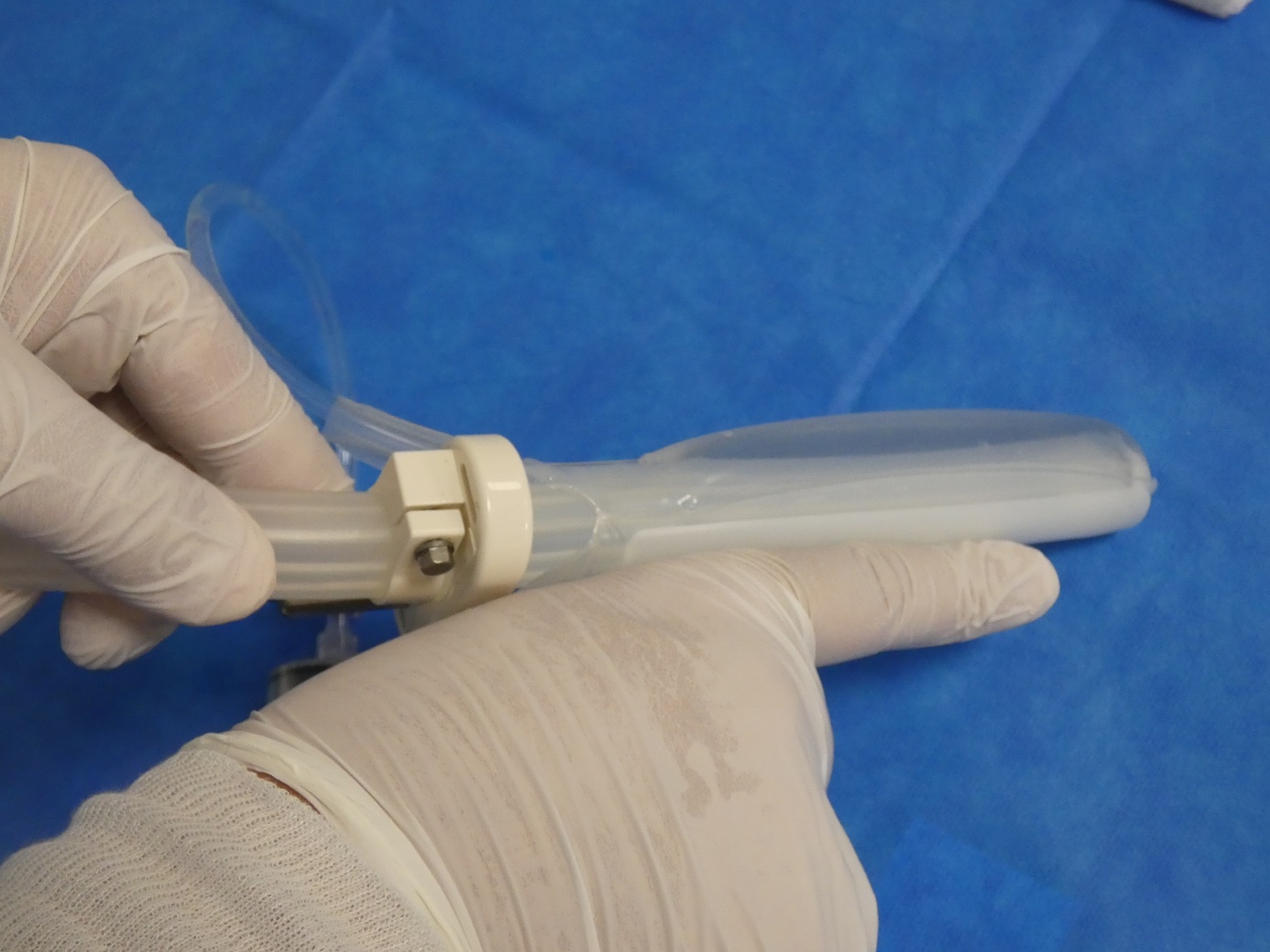
**

**Figure 5:** Positioning of the filled balloon chamber. The filled chamber of the balloon is oriented endorectally toward the non-tumor-bearing rectal wall to protect the mucosa by increasing the distance from the radiation source/rectal applicator. The plastic clamp on the unfilled side of the balloon is in direct contact with the applicator surface and adheres in situ to the tumor-bearing rectal wall. Alignment is based on the tumor localization as described in transverse anatomical reference planes during MRI and rectoscopy, as well as in correlation with direct digital rectal examination, depending on the tumor's height. This illustration provides a preview of the applicator in its final configuration prior to HDR irradiation.

**
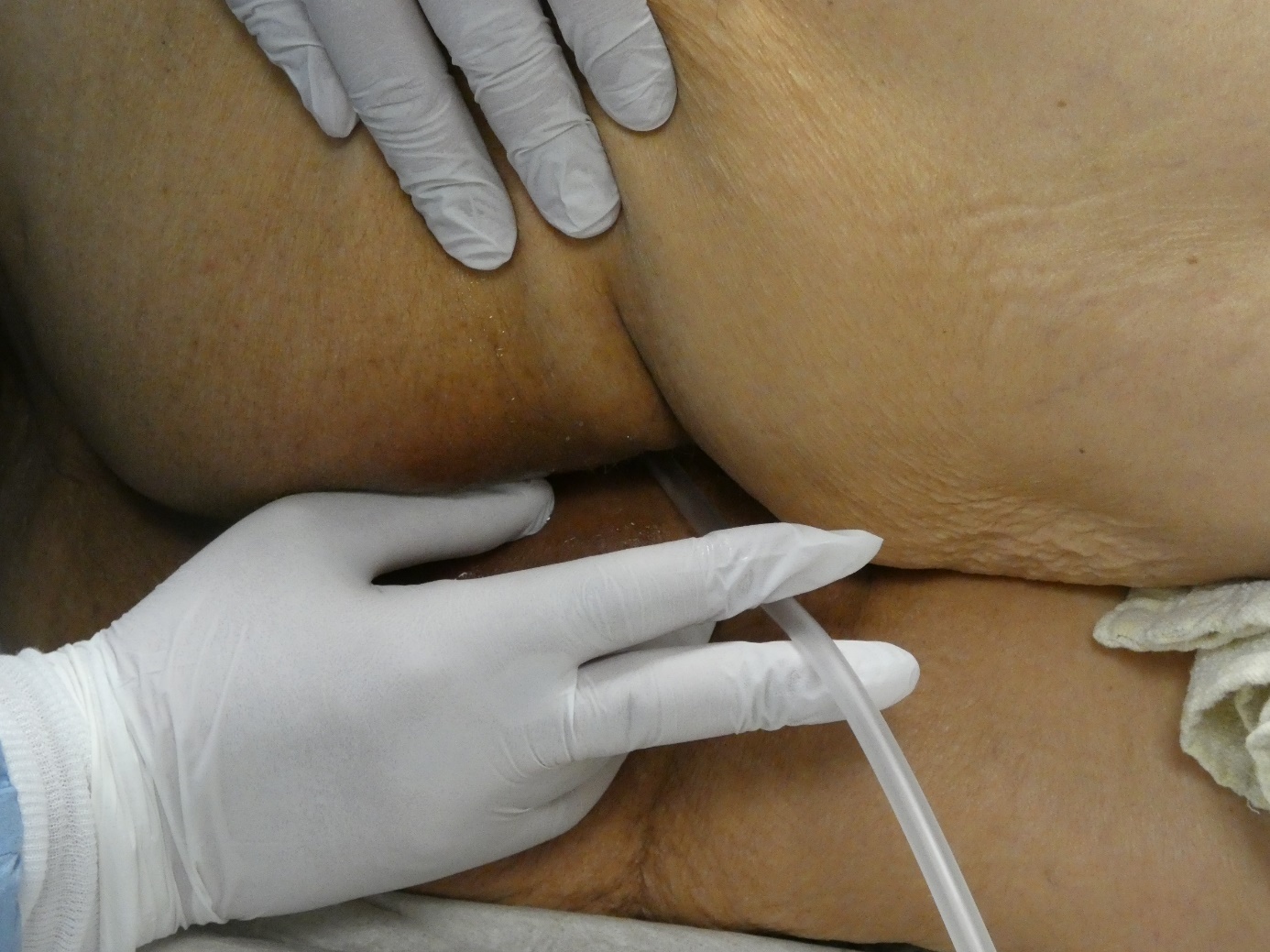
**

**Figure 6: First Step of Treatment:** Patient Positioning in the First Step of Treatment. In the first step of treatment, the patient is positioned on the CT table in the left lateral decubitus position with knees bent. Alternatively, the patient can be placed on a CT-compatible treatment table, which is subsequently moved onto the CT table for 3D treatment planning. Regardless of the table choice, after positioning, a rectal tube is inserted to relieve intestinal gas. An insertion depth of 10–15 cm is recommended.

**
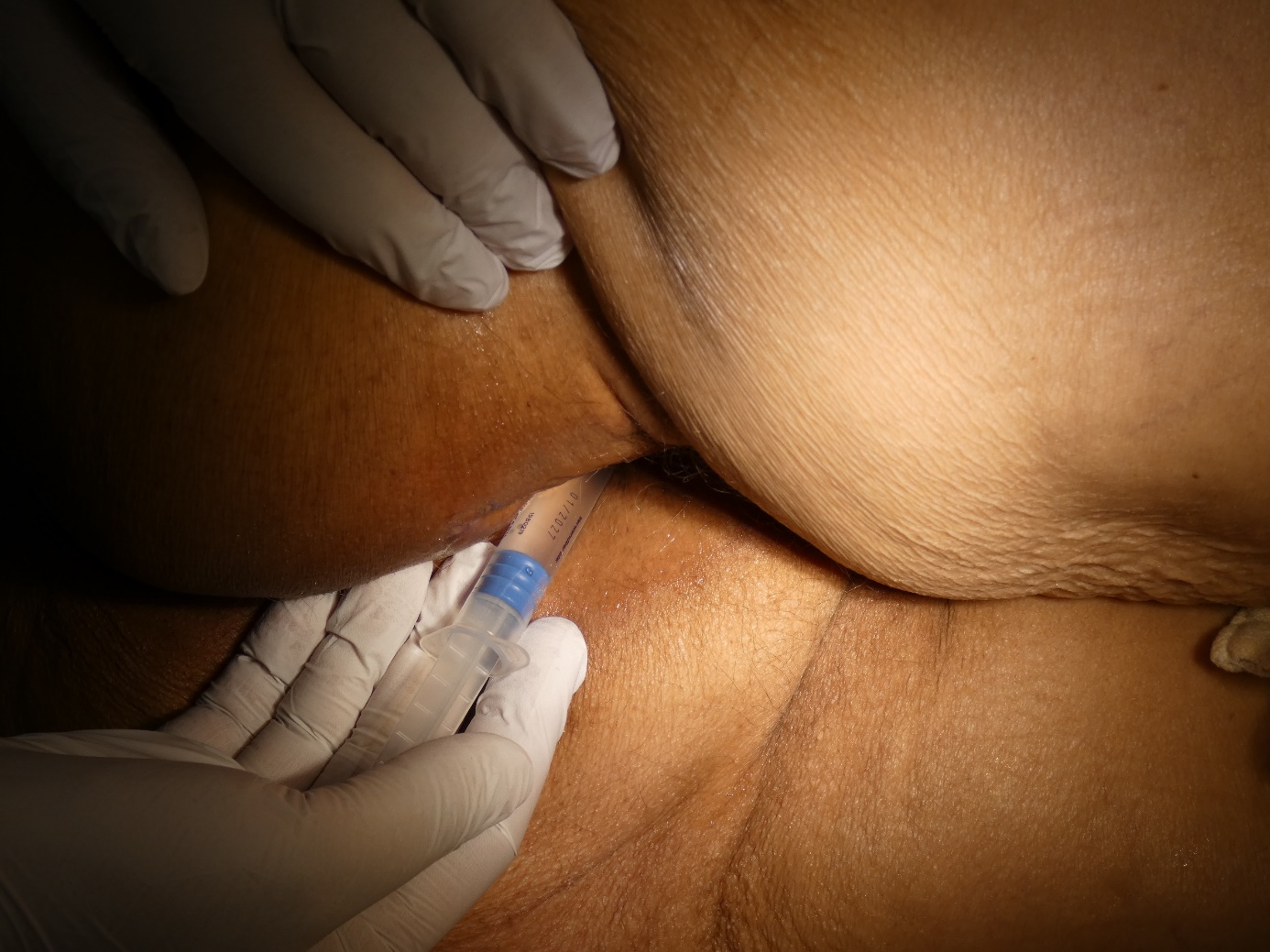
**

**Figure 7: Second step: Deep endorectal instillation of lidocaine gel:**
In the second step, 30 ml of lidocaine-containing lubricating gel (e.g., three 10 ml Instillagel© prefilled syringes) is deeply instilled into the rectum prior to applicator insertion. This procedure is performed with the patient in the left lateral decubitus position with knees bent.

**
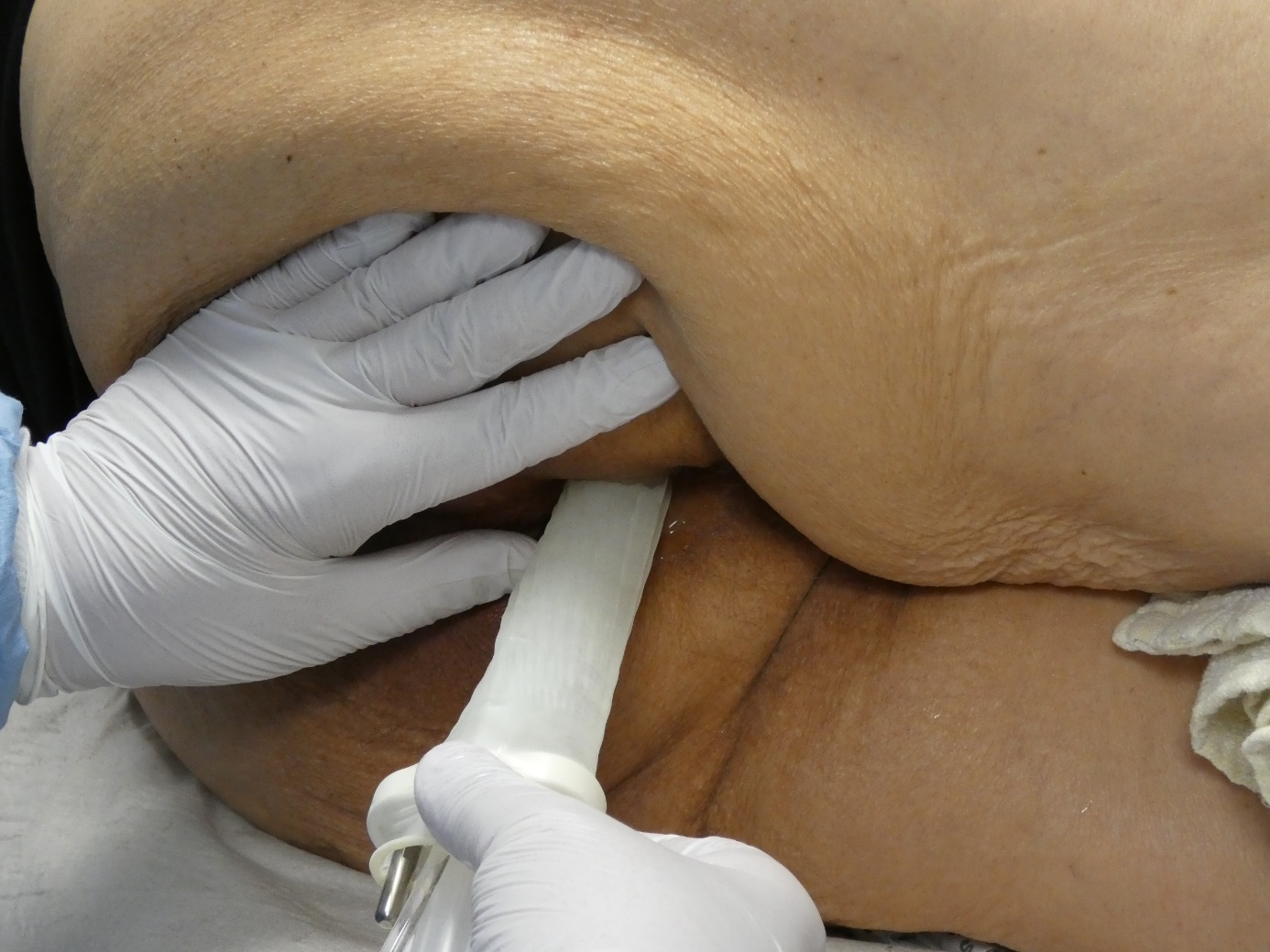
**

**Figure 8: Third step: Insertion of the applicator**. In the third step, the applicator is inserted. Initially, a ventral angulation (toward the bladder) is applied for the first few centimeters after passing the anal sphincter. A protective condom is placed over the applicator with the cover balloon. This procedure is performed with the patient in the left lateral decubitus position with knees bent.


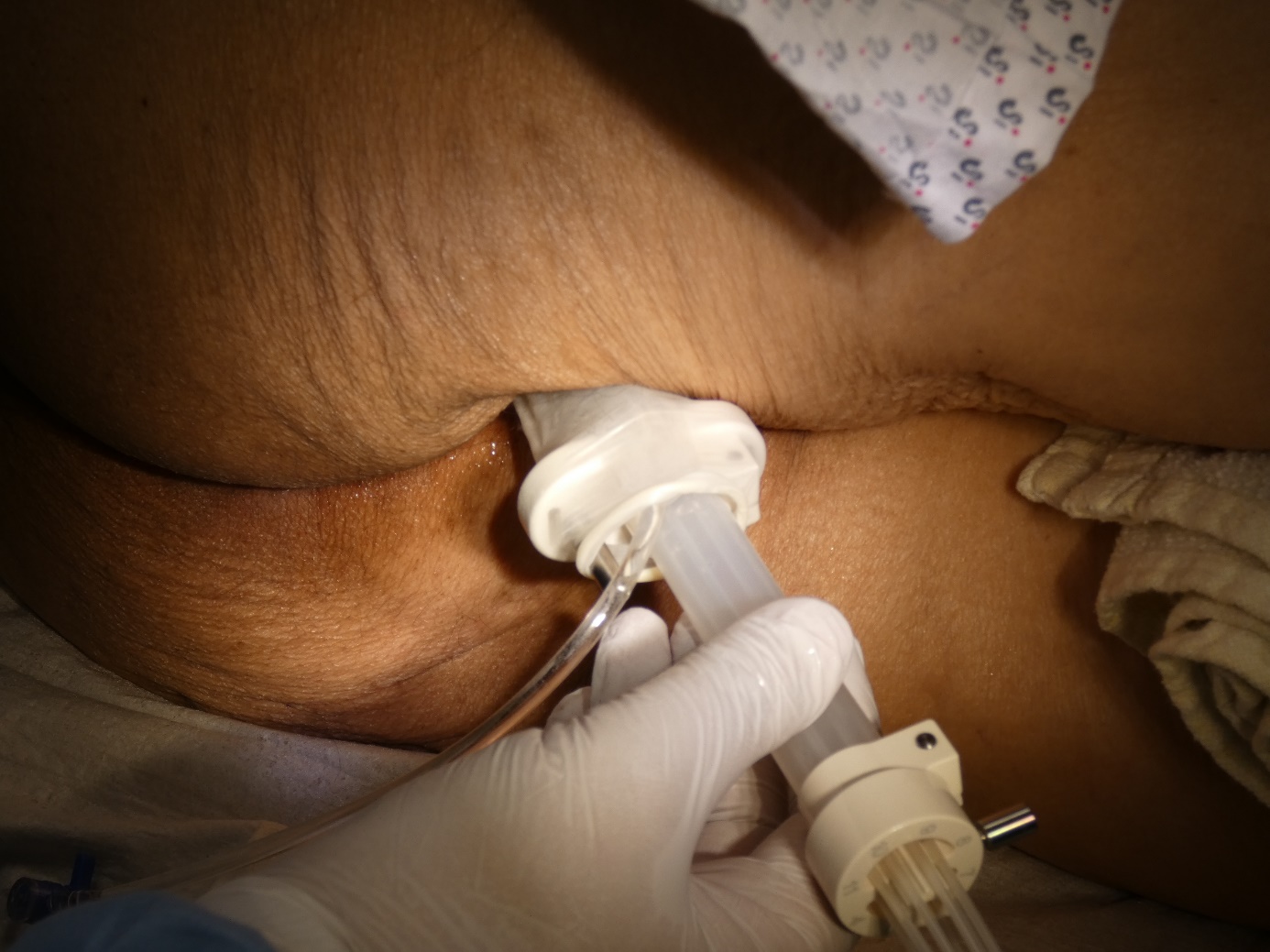


**Figure 9: Fourth step:** Adjustment of applicator angulation. After inserting approximately 5 cm or clearly passing the anal sphincter, the applicator angulation is adjusted dorsally (toward the sacrum) until the final depth is reached. The upper pole (tip) of the applicator should be positioned 10–15 mm deeper than the cranial tumor margin. The documentation of rectoscopy in transverse anatomical reference planes (SSL) is used to determine the appropriate insertion depth. This procedure is performed with the patient in the left lateral decubitus position with knees bent.


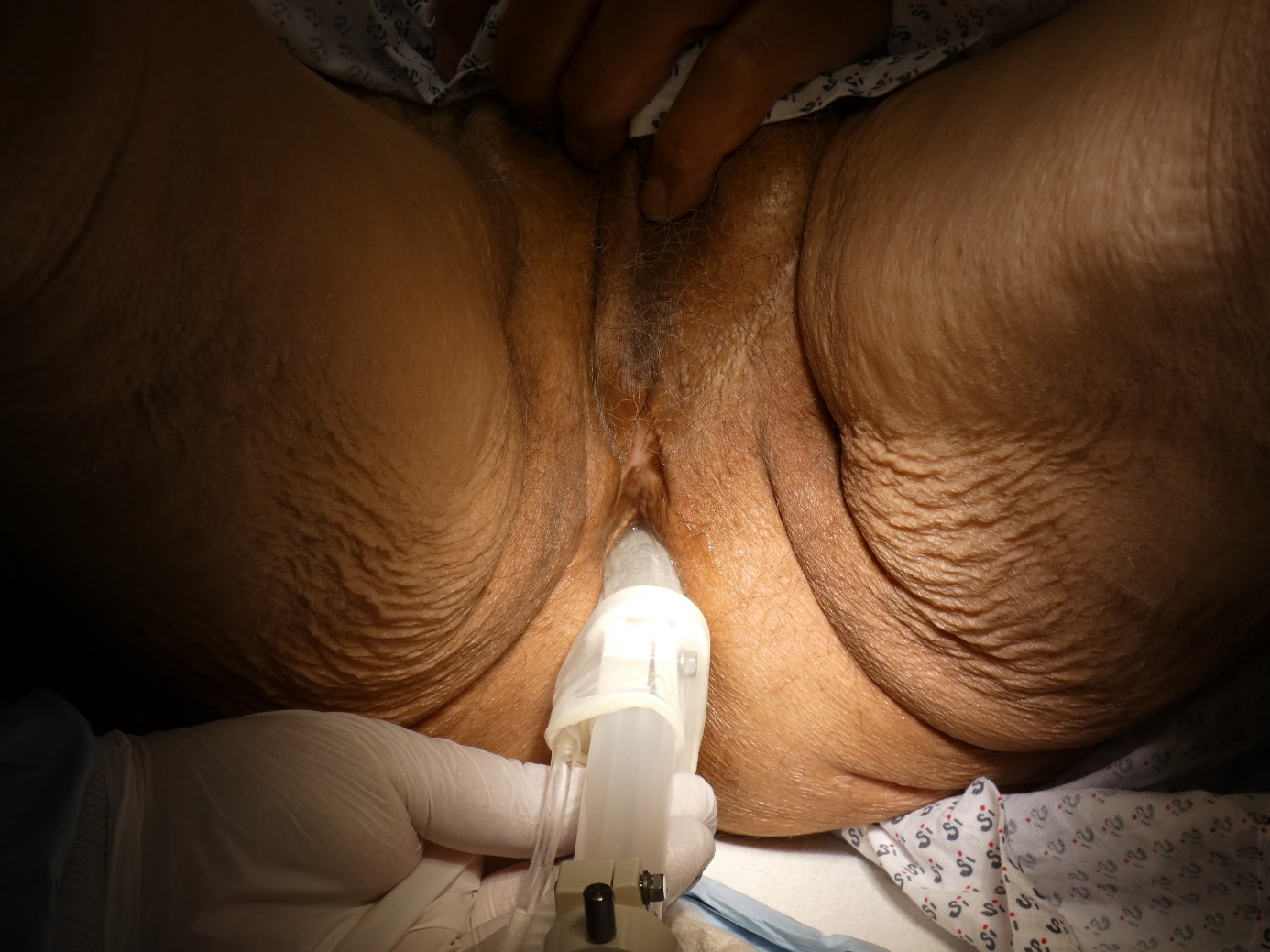


**Figure 10: Fifth step: Transition to supine position.** In the fifth step, the patient is turned to the supine position while manually supporting and maintaining the position of the inserted applicator. Once the patient is fully in the supine position, the knees remain bent.


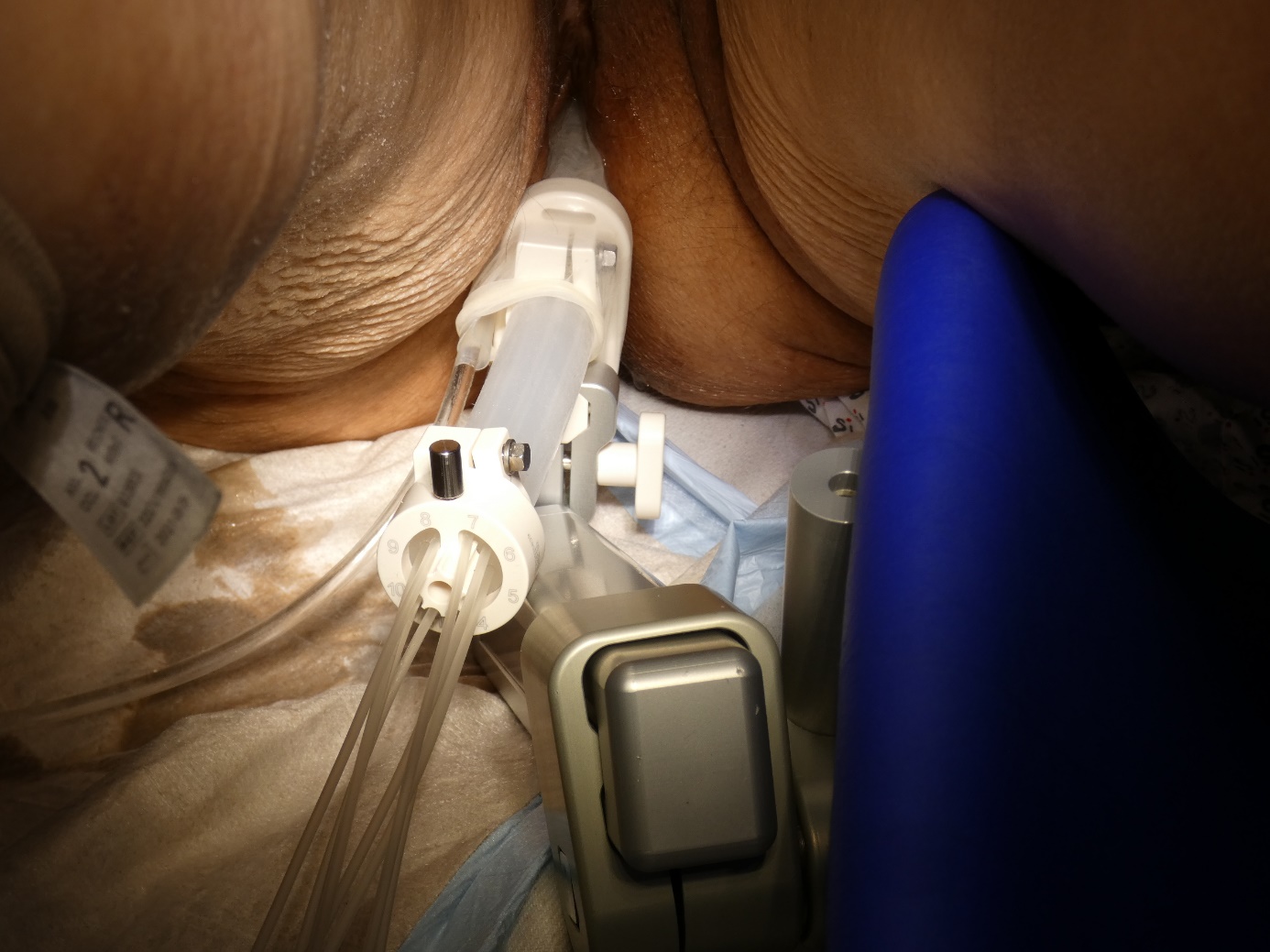


**Figure 11a: Sixth step: Securing the Applicator.** In the sixth step, the applicator is secured using the product-specific fixation device, which is positioned centrally between the patient’s legs to ensure stable endorectal placement. It is essential to maintain a parallel alignment of the applicator relative to the treatment table, which can be achieved using the adjustable features of the fixation device. Afterward, the legs are extended and positioned in foam supports. A Velcro or similar strap is applied at ankle level to stabilize the leg position.


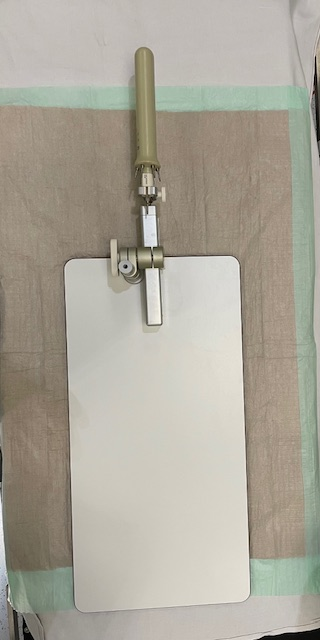

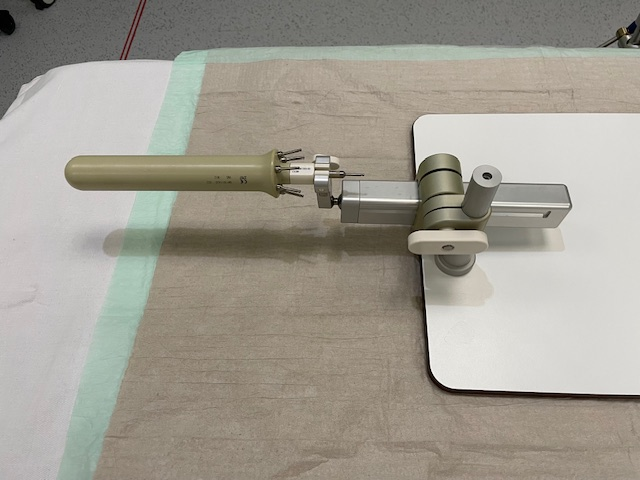


**Figure 11b: Supplemental illustration of MCC fixation (Varian).** This figure provides an additional view of the fixation of the MCC applicator (by Varian) within the holder, demonstrating secure positioning for treatment.


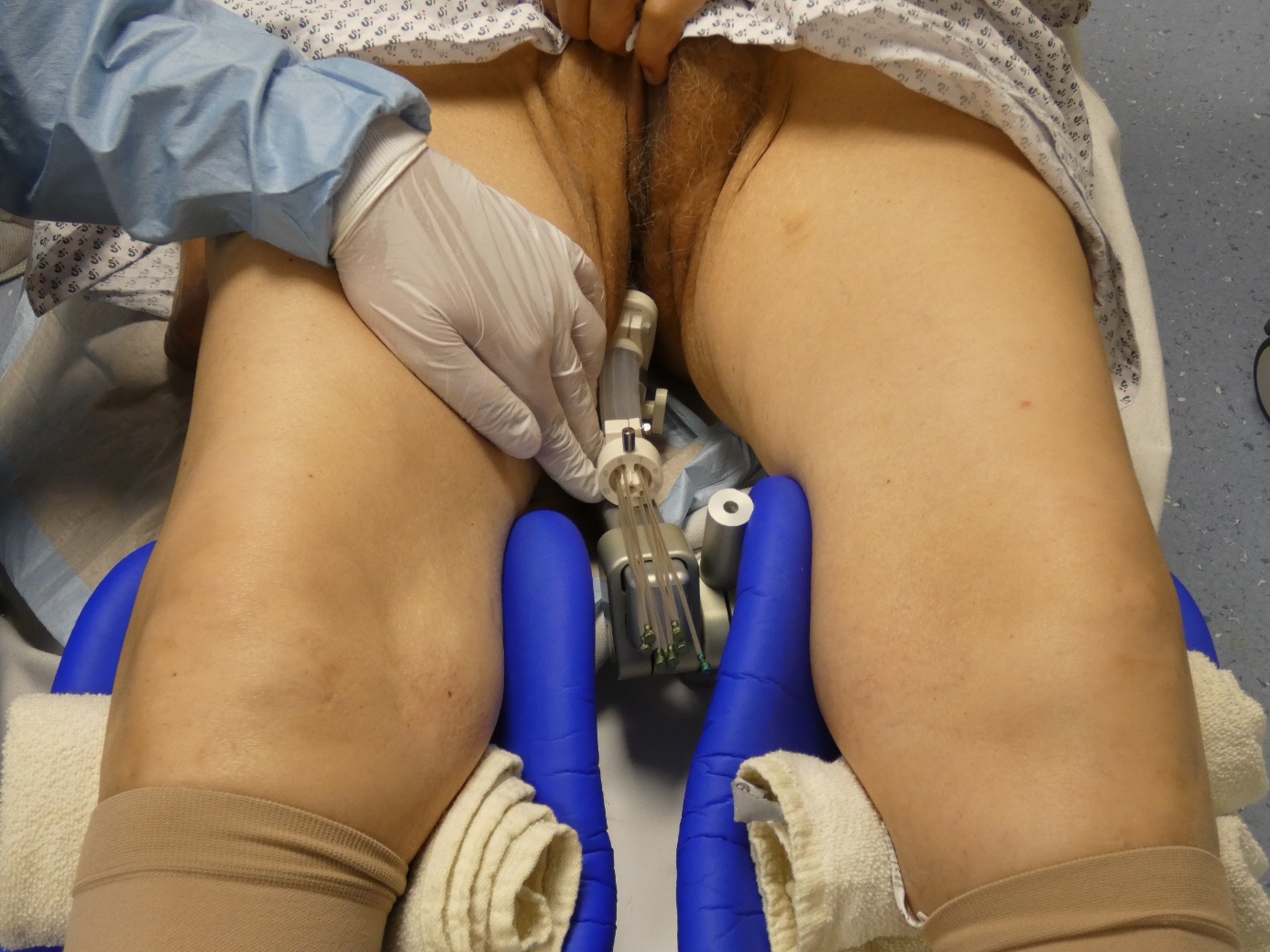


**Figure 12: Seventh step.** Insertion of radiopaque markers. In the seventh step, radiopaque markers are inserted into all channels of the applicator (for the flexible applicator). These markers enable the reconstruction of the channels for 3D treatment planning. The green tips of the markers are visible at the ends of each channel catheter.


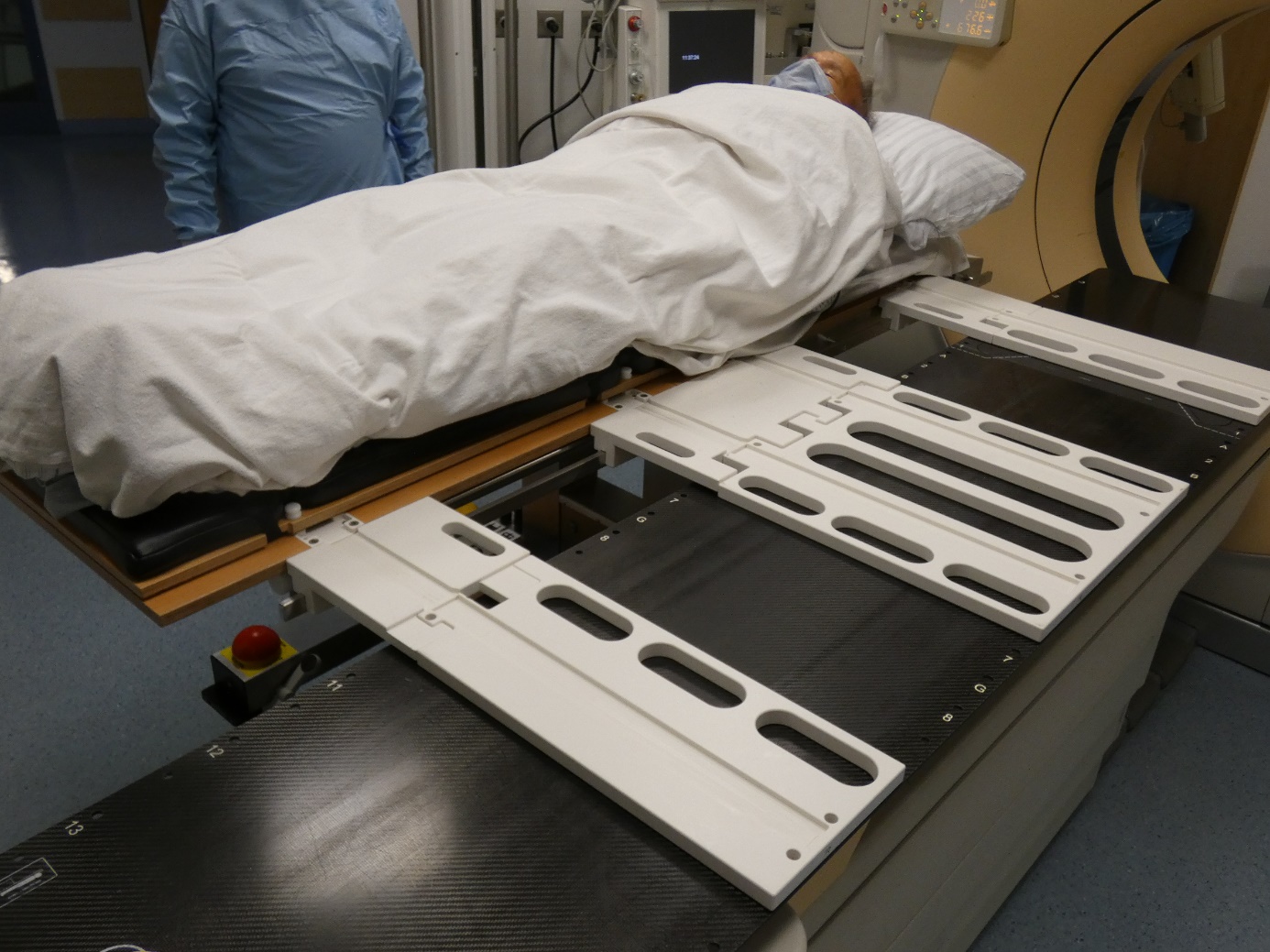


**Figure 13: Eighth Step.** Patient positioning for Planning CT. In the eighth step, the patient is positioned on the planning CT to generate a DICOM dataset for 3D treatment planning. In this example, the patient is placed on a CT-compatible treatment table, which can be moved onto the device-specific CT table without altering the patient’s position. This approach enhances implant stability, as no adjustments are made to the immobilized patient.


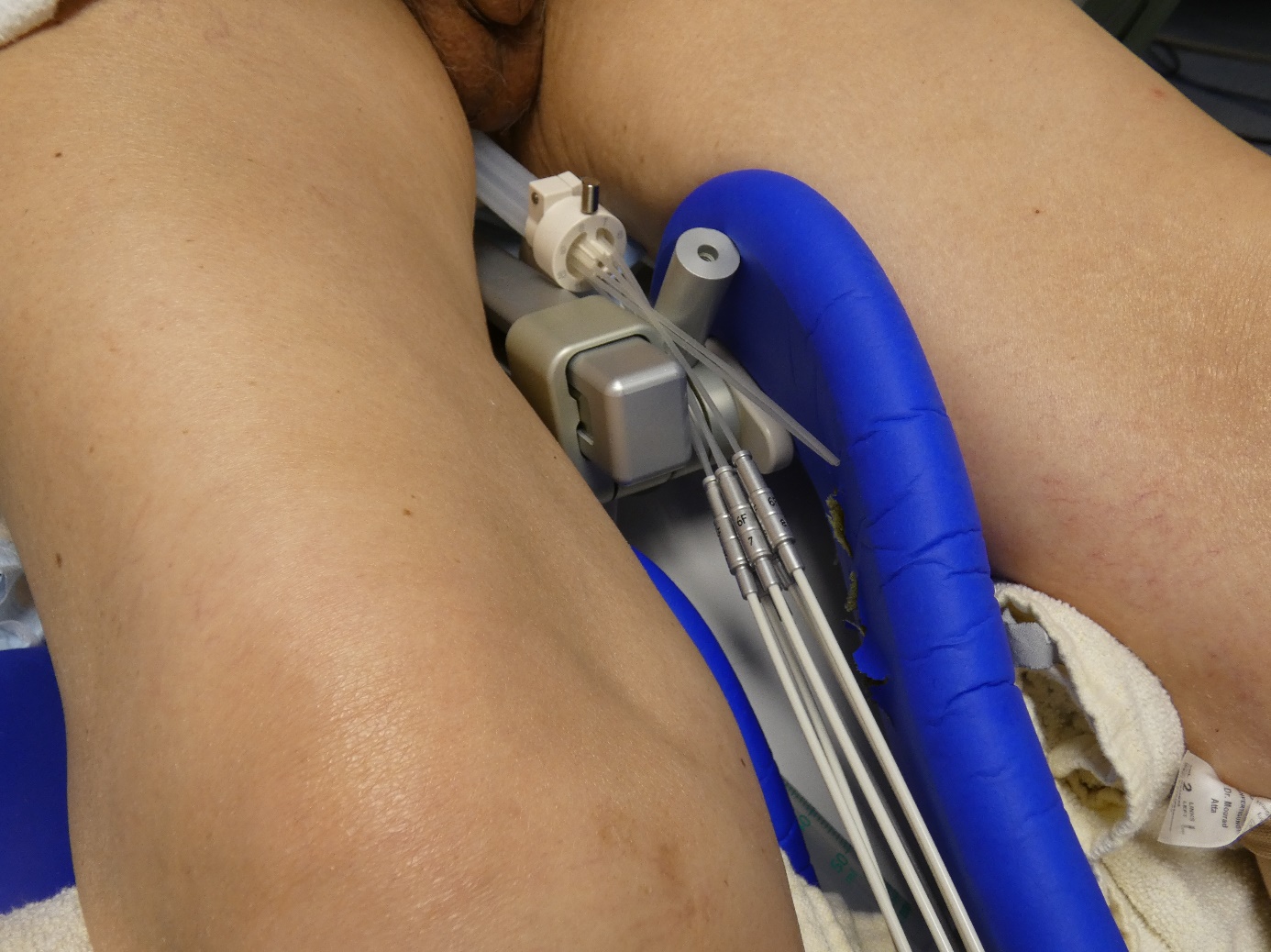


**Figure 14: Ninth Step: Connection of applicator channels to the afterloader**. In the ninth step, after completing 3D treatment planning, the applicator channels are connected to the afterloader using transfer tubes. Only the channels of the multichannel rectal applicator that are loaded to cover the planning target volume (PTV) as determined during treatment planning are connected.


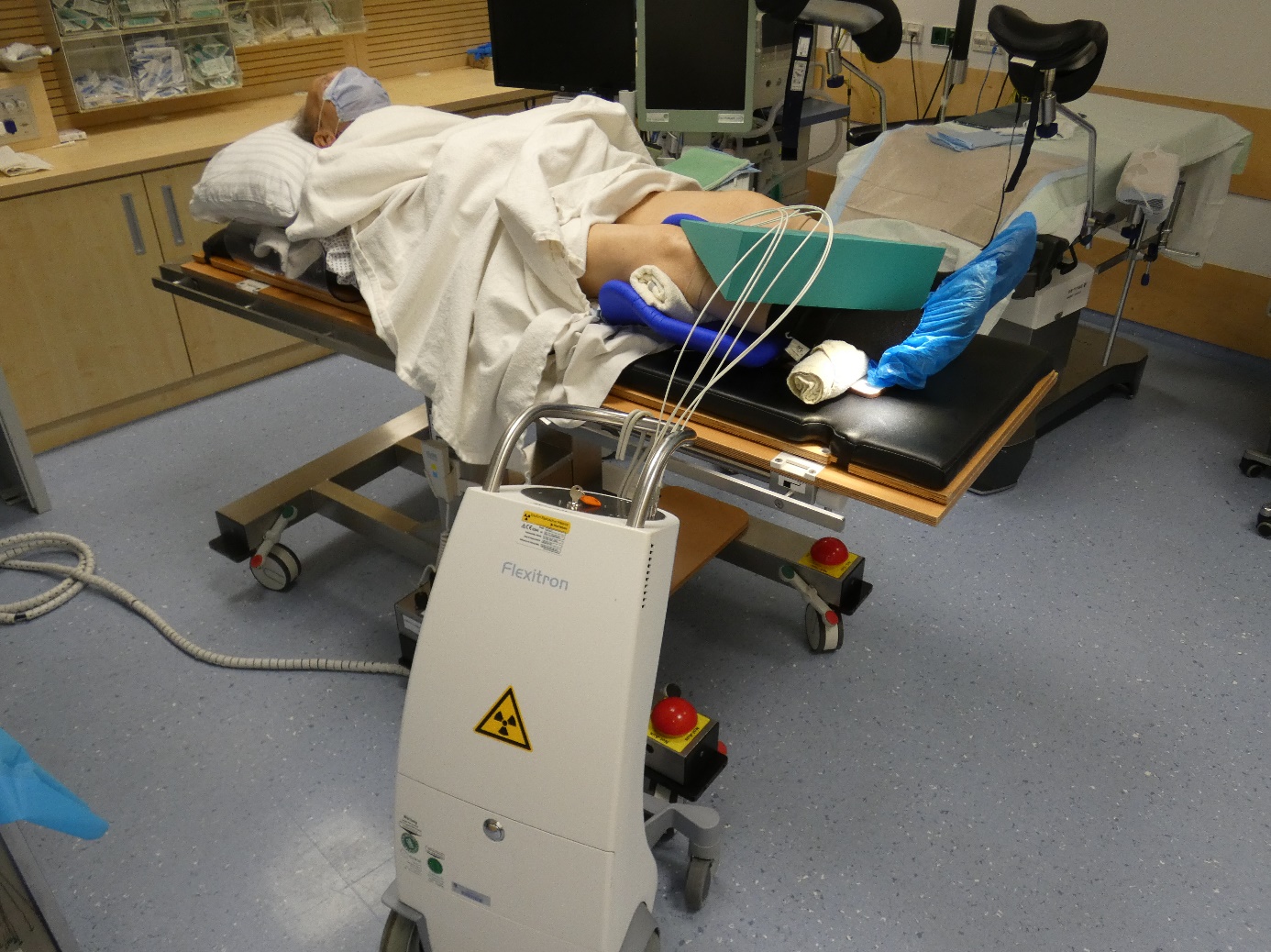


**Figure 15: Tenth Step. Endorectal HDR irradiation with afterloading technique.** In the tenth step, endorectal HDR irradiation is performed using the afterloading technique. The tubing from the afterloader to the patient is distanced from the body (legs) by placing it on foam cushions.


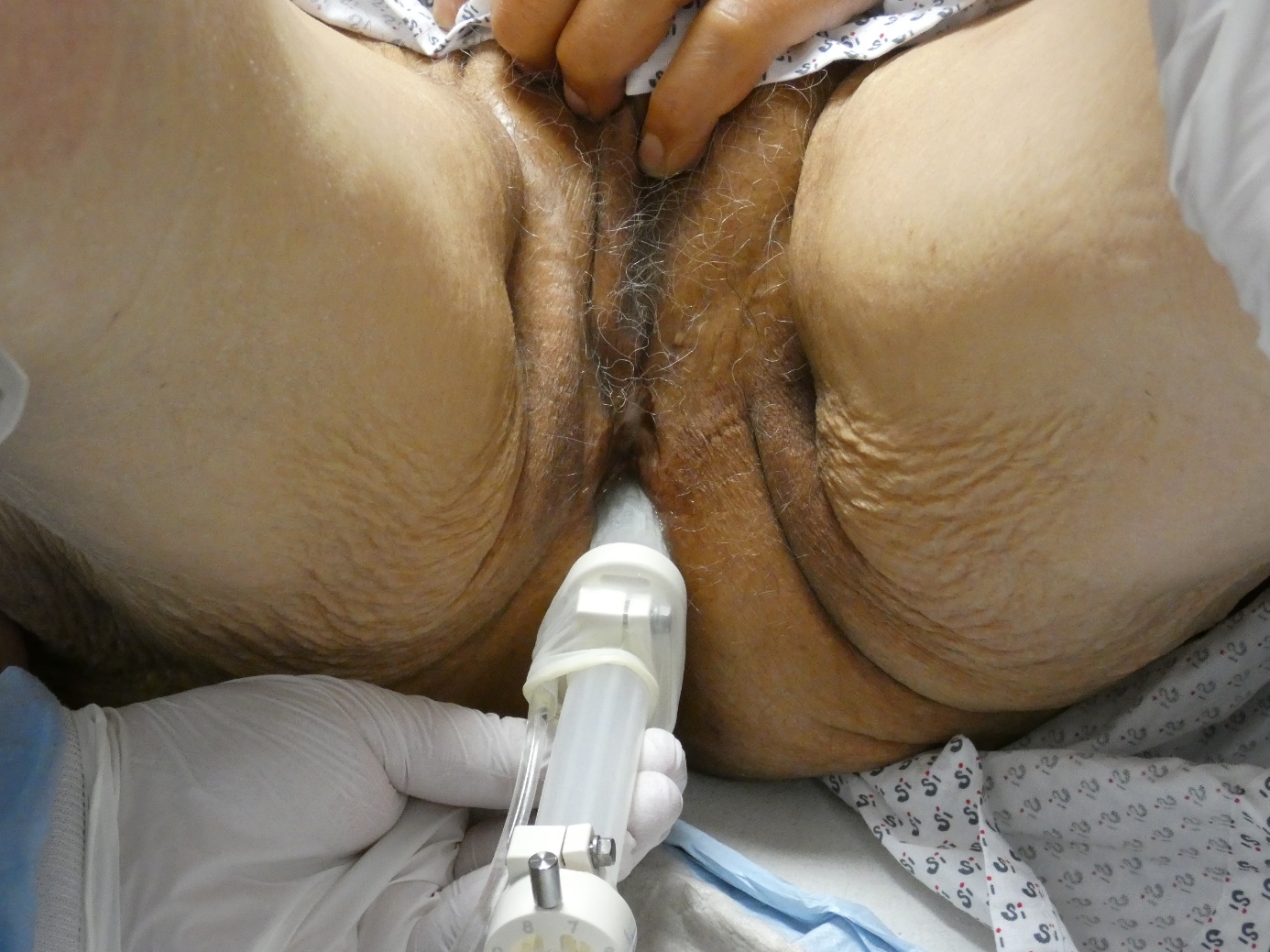


**Figure 16: Eleventh and Final Step. Disconnection and applicator removal.** After completing the irradiation, the afterloader is disconnected, and all tubing is removed. The patient’s legs are bent at the knees, and the applicator is explanted under direct visualization. Following the cleaning of any expelled intestinal contents, the patient is positioned in a seated posture and subsequently transferred to the hospital bed.

**Target Volume Concept and Dose Prescription of brachytherapy**

The organ-preserving treatment strategy involves an EBRT regimen for the pelvis delivered with moderate hypofractionation to a total dose of 39 Gy (5×3 Gy per week) over 3 weeks, in accordance with ICRU 98 guidelines. Following a six-week interval after the completion of EBRT, endorectal high-dose-rate brachytherapy (HDR-BT) is initiated as per the specified protocol.

*Gross Tumor Volume (GTVb):*

The delineation of the macroscopic residual tumor (GTVb) should primarily rely on endoscopic evaluation and the placement of implanted clips. Active involvement of the planning radiation oncologist during endoscopy, or at least a detailed consultation with the endoscopist, is strongly recommended, particularly to confirm the distances between the implanted clips and the tumor the alignment of the clips and their position clockwise. Additionally, imaging modalities such as MRI should be incorporated, as they are essential for assessing tumor depth and intramural extension. Nevertheless, caution must be exercised, as the anatomical conditions visualized on MRI can differ considerably from those seen on CT with an inserted applicator, where rectal wall compression is often observed.

*Clinical Target Volume (CTVb):*

For this study, a standardized assumption of a predefined CTV margin around the GTV to account for microscopic tumor spread is not intended. The **CTVb should effectively align with the GTVb** as all suspected residual tumor areas are included in the GTVb.

*Planning Target Volume (PTVb):*

To account for uncertainties such as delineation inaccuracies (e.g., slice thickness), applicator displacement, or rotation, a PTV margin can be applied. The exact margin should be determined by individual treatment centers, depending on the accuracy of the respective plan, while carefully weighing the potential for increased toxicity, especially if it leads to significant increases in the irradiated circumference or depth prescription. As a general guideline, a margin of approximately 5 mm cranially/caudally and 2 mm circumferentially from the CTV is recommended, excluding OARs, the applicator, balloon, anal canal and rectal lumen.

*Dose Prescription & Treatment Planning:*

**The primary objective of treatment planning is to achieve complete coverage of the CTVb by the 100%-isodose (8 Gy)**, while maintaining a conformal dose distribution, particularly when using a multichannel applicator. It should also be aimed that the PTVb is at least covered by the 75% isodose (6 Gy). To avoid excessive maximum doses at the applicator surface or within the CTV, which could result in ulcerations or high-grade proctitis, **the dose prescription (100% = 8 Gy) should be limited to a maximum depth of 1 cm** (refer to *Constraints for Organs-at-Risk*).

Alternatively, contact X-ray brachytherapy (CXB), delivering 90 Gy in three fractions (30 Gy per fraction), as demonstrated in the OPERA study (Gerard et al., The Lancet Gastroenterology & Hepatology, 2023), may be considered as an alternative to HDR-BT.

**Constraints for Organs-at-Risk and further planning considerations**

Accurate brachytherapy planning and execution are essential for the success of treatment. In addition to achieving significant remission, the avoidance of higher-grade toxicities should always be a priority, and both objectives must be carefully balanced, especially in this vulnerable cohort. This is particularly important because quality of life (a second primary endpoint) is significantly impacted by toxicity.

In some cases, compromises in dose prescription may be necessary to prevent severe toxicities. This is especially relevant for extensive carcinomas, tumors involving large portions of the circumference, those with significant depth of invasion, or patients on anticoagulation therapy.

The primary organ at risk (OAR) during HDR brachytherapy (HDR-BT) is the rectal wall, which overlaps with the clinical target volume (CTV). This means the CTV simultaneously represents both the target and the OAR. Garant et al. recommended a dose constraint of 3×20 Gy to the surface, which was associated with acceptable toxicity levels. In the HERBERT-I study, a CTV D2cc <14 Gy per fraction was proposed to minimize the risk of rectal ulceration. **Therefore, a CTV D2cc <14 Gy per fraction is a mandatory constraint for this study** (even if the intended CTVb coverage cannot be fully achieved as a result)**.**

Other OARs include the rectal wall outside the CTV (i.e., the normal rectal wall), the anus, bladder, genitals, and bowel loops. However, practical recommendations for constraints on these OARs in patients receiving HDR-BT for rectal cancer are largely lacking. In the HERBERT-I study, hotspots in surrounding organs (D0.1cc > 100%) were not permitted, and no toxicity was observed in the anus, vagina, or small bowel. The documented toxicity to the normal rectal wall underscores the importance of “shielding” the wall to better preserve normal tissue and should therefore be strongly pursued.

Special care is required when irradiating distal rectal cancers to avoid proctitis. In the HERBERT-I study, the EQD2α/β3 did not exceed 66 Gy, and no anal toxicity was observed. For the bladder and genitals as OARs, clinical experience from prostate and gynecological brachytherapy can be applied.

It is essential to inform patients who require long-term anticoagulation therapy about the increased risk of rectal bleeding, both as an acute and late complication following HDR-BT.

In particular, a comprehensive analysis of data from the MORPHEUS Phase II–III Study and HERBERT I studies highlights that not only the surface dose but also the intratumoral dose gradient, the sparing of unaffected rectal mucosa, and adaptive planning are crucial to minimizing high-grade toxicities. Auxiliary techniques such as a double-balloon approach (flattening the dose within the target volume), a shielded applicator, or adaptive planning can help optimize the balance between achieving maximum dose coverage and limiting doses to avoid toxicities.

**Supportive Measures**

- Prevention of High-Grade Peri-Interventional Toxicity. To prevent high-grade peri-interventional toxicity during the 3 weeks of brachytherapy, instillation with glucocorticoid foam (e.g., Budesonide) should be administered twice weekly.
- Management of Early or Late Manifest Toxicity. For cases of manifest early or late toxicity, conservative treatment primarily involves the instillation of glucocorticoid foam (e.g., Budesonide) three times per week. This treatment can be continued for up to 3 months under regular clinical monitoring.
- Persistent Symptoms and High-Grade Rectitis/Proctitis. In cases of persistent symptoms or high-grade rectitis/proctitis with rectal bleeding, Argon Plasma Laser treatment may be considered, based on existing literature. Risk factors for persistent bleeding or ulceration include complete clinical remission with crater formation following EBRT and the use of full anticoagulation therapy.
- Boost Dose in Complete Remission. In patients with complete remission (confirmed by MRI and endoscopy) following EBRT, a boost dose of 8 Gy to a depth of 5 mm can be considered.

**REFERENCES**

[1] Garant A, Vasilevsky CA, Boutros M, Khosrow-Khavar F, Kavan P, Diec H, et al. MORPHEUS Phase II-III Study: A Pre-Planned Interim Safety Analysis and Preliminary Results. Cancers (Basel). 2022;14.

[2] Garant A, Magnan S, Devic S, Martin AG, Boutros M, Vasilevsky CA, et al. Image Guided Adaptive Endorectal Brachytherapy in the Nonoperative Management of Patients With Rectal Cancer. Int J Radiat Oncol Biol Phys. 2019;105:1005-11.

[3] Devic S, Bekerat H, Garant A, Vuong T. Optimization of HDRBT boost dose delivery for patients with rectal cancer. Brachytherapy. 2019;18:559-63.

[4] Rijkmans EC, Cats A, Nout RA, van den Bongard D, Ketelaars M, Buijsen J, et al. Endorectal Brachytherapy Boost After External Beam Radiation Therapy in Elderly or Medically Inoperable Patients With Rectal Cancer: Primary Outcomes of the Phase 1 HERBERT Study. Int J Radiat Oncol Biol Phys. 2017;98:908-17.

[5] Fleischmann M, Diefenhardt M, Trommel M, Scherf C, Ramm U, Chatzikonstantinou G, et al. Image-guided high-dose-rate brachytherapy for rectal cancer: technical note and first clinical experience on an organ-preserving approach. Strahlenther Onkol. 2022;198:654-62.

[6] Fokas E, Glynne-Jones R, Fleischmann M, Piso P, Tselis N, Ghadimi M, et al. Radiotherapy dose escalation using endorectal brachytherapy in elderly and frail patients with rectal cancer unsuitable for surgery: Lessons from studies in fit patients and future perspectives. Cancer Treat Rev. 2022;112:102490.

[7] Gerard JP, Barbet N, Schiappa R, Magne N, Martel I, Mineur L, et al. Neoadjuvant chemoradiotherapy with radiation dose escalation with contact x-ray brachytherapy boost or external beam radiotherapy boost for organ preservation in early cT2-cT3 rectal adenocarcinoma (OPERA): a phase 3, randomised controlled trial. Lancet Gastroenterol Hepatol. 2023;8:356-67.

# Follow-up schedule in case of surgery

The ACO/ARO/AIO-22 clinical trial will only include elderly and frail patients unfit for surgery. Upon completion of HDR-BT restaging with pelvic MRI and endoscopy will be conducted 12 weeks after the last HDR-BT application. Patients with poor response or, even, tumor progression will be offered palliative care according to the discretion of the treating physician, including re-evaluation of the patient operability. ***Nevertheless,* if patients will be considered operable at 6 months after treatment initiation or in case of tumor regrowth (despite initially found to be unsuitable for surgery), then follow-up should be performed as shown in the Table 1 below**. Follow-up procedures and intervals in this case of curative (R0) radical surgery (TME) or LE/TEM (or salvage surgery for local regrowth) are according to the “S3-Leitlinien der Deutschen Krebsgesellschaft”.

**Table 1.** Follow-up schedule after decision for palliative care or surgery

| **Months after surgery** | **3** | **6** | **12** | **18** | **24** | **36** | **48^1^** | **60^1^** |
| --- | --- | --- | --- | --- | --- | --- | --- | --- |
| Physical examination, tumor marker CEA |  | X | X | X | X | X | X | X |
| Colonoscopy* |  | X* |  |  |  |  |  | X |
| Abdomen sonography |  | X | X | X | X | X | X | X |
| Rectoscopy** |  | X | X | X | X |  |  |  |
| Spiral computer tomography*** | X |  |  |  |  |  |  |  |
| Chest X-ray |  |  | X |  | X | X | X | X |

* if a colonoscopy was not performed before surgery; next colonoscopy in 5 years in cases of normal findings (lack of adenoma or carcinoma); ** the S3 guidelines recommend rectoscopy only in case of rectal cancer without neoadjuvant/adjuvant chemoradiation, however, within this prospective study, rectoscopy is recommended especially in case of local excision as surgical procedure; *** 3 months after completion of tumor specific therapy (TNT+sugery) as baseline postoperative imaging; ^1^ according to the “S3-Leitlinien der Deutschen Krebsgesellschaft”.
